# Non-canonical substrate recognition by the human WDR26-CTLH E3 ligase regulates prodrug metabolism

**DOI:** 10.1016/j.molcel.2024.04.014

**Published:** 2024-05-16

**Authors:** Karthik V. Gottemukkala, Jakub Chrustowicz, Dawafuti Sherpa, Sara Sepic, Duc Tung Vu, Özge Karayel, Eleftheria C. Papadopoulou, Annette Gross, Kenji Schorpp, Susanne von Gronau, Kamyar Hadian, Peter J. Murray, Matthias Mann, Brenda A. Schulman, Arno F. Alpi

**Affiliations:** 1 Department of Molecular Machines and Signaling, Max Planck Institute of Biochemistry, Martinsried 82152, Germany; 2 TUM School of Natural Sciences, Technical University, Munich 85748, Germany; 3 Department of Proteomics and Signal Transduction, Max Planck Institute of Biochemistry, Martinsried 82152, Germany; 4 Immunoregulation, Max Planck Institute of Biochemistry, Martinsried 82152, Germany; 5 Research Unit-Signaling and Translation, Cell Signaling and Chemical Biology, Helmholtz Zentrum München, Neuherberg 85764, Germany

## Abstract

The yeast GID E3 ubiquitin ligase forms a suite of complexes with interchangeable receptors that selectively recruit N-terminal degron motifs of metabolic enzyme substrates. The orthologous higher eukaryotic CTLH E3 complex has been proposed to also recognize substrates through an alternative subunit, WDR26, which promotes formation of supramolecular CTLH E3 assemblies. Here, we discover that human WDR26 binds the metabolic enzyme NMNAT1 and mediates its CTLH E3-dependent ubiquitylation independently of canonical GID/CTLH E3-family substrate receptors. The CTLH subunit YPEL5 inhibits NMNAT1 ubiquitylation and cellular turnover by WDR26-CTLH E3, thereby affecting NMNAT1-mediated metabolic activation and cytotoxicity of the prodrug tiazofurin. Cryo-EM structures of NMNAT1- and YPEL5-bound WDR26-CTLH E3 assemblies reveal an internal basic degron motif of NMNAT1 essential for targeting by WDR26-CTLH E3, and degron mimicry by YPEL5’s N-terminus antagonizing substrate targeting. Thus, our data provides mechanistic understanding of how the YPEL5-WDR26-CTLH E3 acts as a modulator of NMNAT1-dependent metabolism.

## Introduction

Selectivity by the Ubiquitin Proteasome System (UPS) is primarily determined by numerous E3 ubiquitin ligases that mark proteins with ubiquitin recognized by the 26S proteasome.^1,2^ Substrates for ubiquitylation display specific degradation signal motifs - known as degrons - which are recognized by substrate receptors.^3–7^ The relative positioning of these receptors and the catalytic modules within the E3 ligases enables ubiquitin transfer onto the recruited substrate. A large number of human E3 ubiquitin ligases, including cullin RING E3 ligases (CRLs) and the APC/C E3 complex,^8–11^ assemble multi-subunit complexes to exchange degron-selective substrate receptors. These interchangeable substrate receptors usually utilize a common docking site harbored by an adaptor subunit of the E3 complex, thereby expanding the repertoire of protein substrates for ubiquitin targeting by a single E3 ligase activity. The assembly of substrate-selective E3 complexes is dynamic and often determined by the conditional availability of a given substrate receptor. In addition, substrate receptor recruitment can be regulated by the selective activation of E3s via posttranslational modification or by specific exchange factors.^12–19^ Despite these advances, our knowledge of alternative modes of substrate regulation by multisubunit E3 ligase complexes remains rudimentary.

A paradigm for interchangeable substrate receptor-regulated E3s is the budding yeast N-degron-pathway Glucose Induced Degradation Deficient (GID) E3 ligase complex. The GID E3 complex employs a suite of interchangeable “Gid4-family” substrate receptors (Gid4, Gid10, and Gid11) to target a variety of metabolic substrate proteins by recognizing their N-terminal degrons.^20–25^ In particular, Gid4 recognizes sequence motifs with N-terminal proline to target gluconeogenic enzymes when changes in metabolic conditions render them superfluous. In a similar way, Gid10 recognizes the N-terminal proline degron of its only known substrate, Art2, thereby regulating amino acid metabolism.^21^ Although not yet reconstituted biochemically, yeast Gid11 has been implicated in recognizing N-terminal threonine of numerous metabolic regulators involved in amino acid and nucleotide biosynthesis.^22^ In addition, GID’s substrate selectivity is modulated by the subunit Gid7 serving as an adaptor that mediates the formation of a 1.5 MDa supramolecular GID E3 assembly. The Gid7-containing complex was named “Chelator-GID” E3 due to its architecture: a large hollow oval with two opposing active sites and two Pro/N-degron binding Gid4 subunits. The Chelator-GID E3 avidly binds and encapsulates the oligomeric gluconeogenic enzyme Fbp1.^25,26^

Many GID E3 subunits are conserved across higher eukaryotic species, which might imply similar function and regulation.^27,28^ However, for the orthologous human CTLH E3 complex, which is involved in a plethora of biological processes,^27,29–31^ it is surprising that only one Gid4-family substrate receptor (human GID4) has been identified.^25,32–34^ Several recent proteomics studies identified candidate CTLH substrates, including GID4-specific substrate binders,^35–38^ however, their selective recognition, ubiquitin targeting by the CTLH E3 complex, and/or functional roles in biological pathways remain largely elusive. Moreover, considering the functions of all the characterized yeast GID E3 complexes, it is perplexing that no metabolic enzymes have yet been described as direct substrates of the human CTLH E3, raising the question of functional resemblance across evolution.

Despite apparent similarities across the GID/CTLH E3 ligase family, emerging data have raised the possibility of alternative mechanisms of substrate recognition in higher eukaryotes. The genome of *D. melanogaster* neither encodes orthologs of yeast Gid4/Gid10/Gid11 substrate receptors, nor their docking subunit Gid5.^28^ Nevertheless, the CTLH E3 is essential in targeting RNA-binding proteins in fly development.^39,40^ Intriguingly, the CTLH E3 complexes in fly and humans have been proposed to employ the Gid7 orthologs, WDR26 and MKLN1, as non-canonical CTLH substrate receptors.^39–42^ Roles of human WDR26 are of particular importance due to its mutation causing developmental disorders.^29^ However, prior structural studies confirmed that the human WDR26 acts as a supramolecular assembly factor similar to yeast Gid7 in allowing formation of higher order complexes similar to yeast Chelator-GID E3.^25,43–46^ Thus, how WDR26 can fulfill being a substrate receptor and as a WDR26-CTLH supramolecular assembly module, remain unclear.^25,44–46^

Here, we report an unanticipated mechanism of substrate recognition by the human WDR26-CTLH E3 supramolecular assembly that is distinct from the canonical yeast GID E3 in many ways, including an independence of a Gid4-family substrate receptor. We identify the metabolic enzyme nicotinamide/nicotinic-acid-mononucleotide-adenylyltransferase 1 (NMNAT1) as a WDR26-dependent, but GID4-independent CTLH substrate. Cryo-EM structures reveal that WDR26 acts as a non-interchangeable substrate receptor for NMNAT1. Despite the overall similar catalytic architecture to that of yeast Chelator-GID E3, whereby the oligomeric substrate is encapsulated in the hollow oval E3, the WDR26-CTLH E3 complex recognizes the hexameric NMNAT1 substrate by its internal KR-rich basic motif. This is additionally regulated by degron mimicry at the N-terminus of another CTLH subunit, YPEL5, antagonizing NMNAT1 ubiquitin targeting by the WDR26-CTLH complex. Consistently, absence of the inhibitory YPEL5 destabilizes cellular NMNAT1. Altogether, these findings provide the molecular basis for a distinct mode of a human CTLH E3 to selectively bind the metabolic substrate NMNAT1.

## Results

### Ubiquitome-enrichment proteomics reveals a CTLH E3 substrate

**Figure 1:**
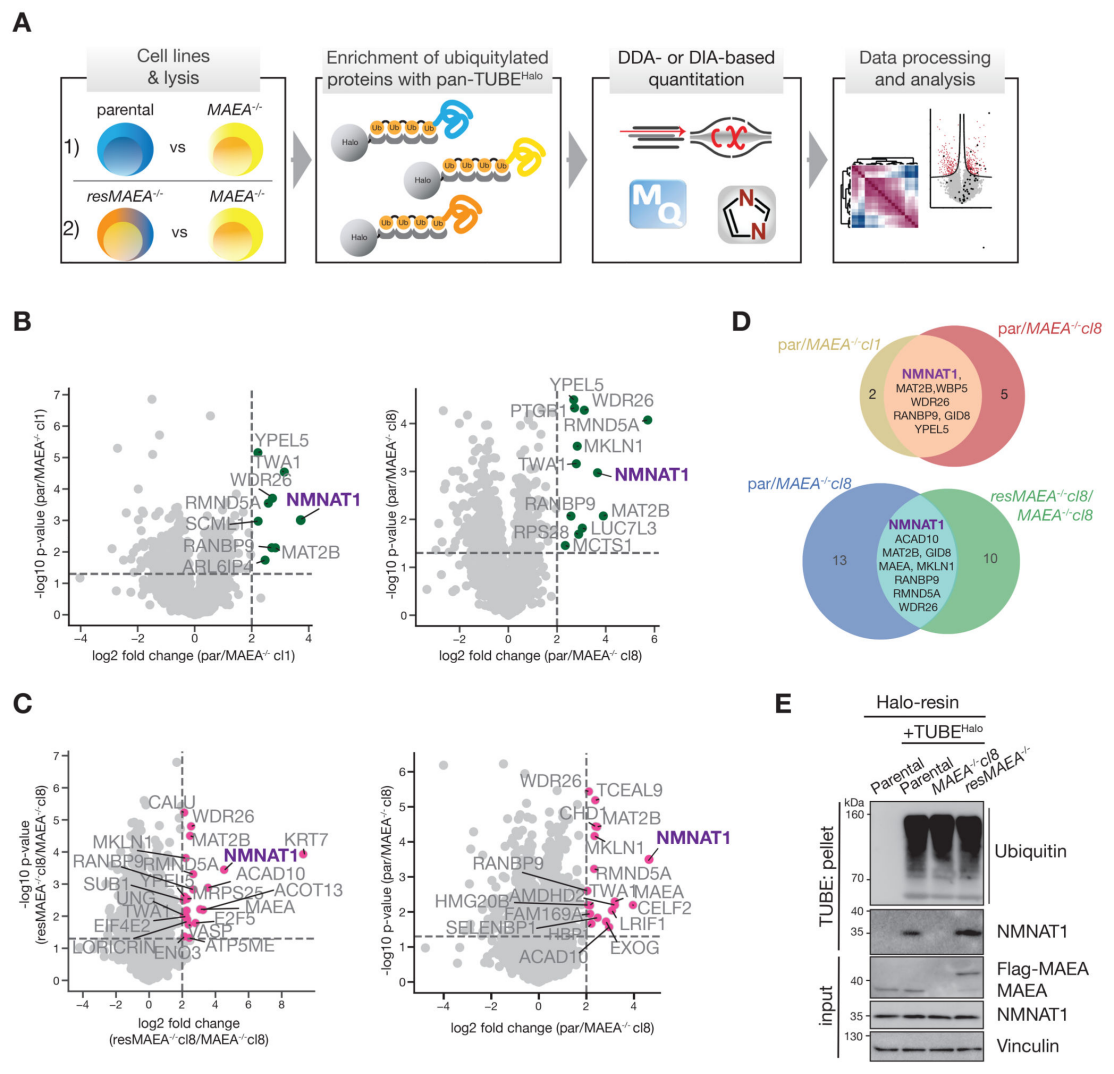
Ubiquitome-enrichment proteomics reveals a CTLH E3 substrate A) Workflow of the proteomics study indicating the used HEK293 cell systems (1) parental vs *MAEA*^*-/-*^ knockout and (2) *MAEA*^*-/-*^ + *MAEA* rescue line (*resMAEA*^*-/-*^) vs *MAEA*^*-/-*^, enrichment of ubiquitylated proteins with Halo-tagged pan-TUBE (TUBE^Halo^), followed by DDA- or DIA-quantitation and data processing/analysis. B) Individual volcano plots of the -log10 p-values vs the log2 protein abundance of TUBE-precipitates between parental and two *MAEA*^*-/-*^ clones (cl1 and cl8). 5% p-value and 4x cut off are indicated. C) Individual volcano plots of the -log10 p-values vs the log2 protein abundance of TUBE-precipitates between *resMAEA*^*-/-*^ vs *MAEA*^*-/-*^*cl8* and between parental vs *MAEA*^*-/-*^ *cl8*. 5% p-value and 4x cut off are indicated. D) Venn diagrams showing overlapping proteins from 5% p-value and 4x cut off of B and C. E) TUBE^Halo^-captured proteins from *MAEA*^*-/-*^*cl8* and *resMAEA*^*-/-*^ cell lines were precipitated with Halo-resin (TUBE: pellet) and analyzed by immunoblot analysis. Vinculin serves as protein input control.

To identify CTLH E3 targets (Figure 1A), we first generated HEK293 cell lines deficient for the catalytic activity of all CTLH E3 assemblies, and a rescue control.^44,47^ CRISPR-Cas9 genome editing was employed to generate loss-of-function alleles in *MAEA* (*MAEA*^*-/-*^), which encodes the catalytic subunit. The stable re-introduction of *MAEA* yielded a rescue cell line (*resMAEA*^*-/-*^) (Figure S1A, S1D). Next, ubiquitin-associated proteins (the “ubiquitome”) were captured from lysates by Halo-tagged Tandem Ubiquitin Binding Entities (TUBEs)^48^ and analyzed using two different mass spectrometry acquisition modes. Data-dependent acquisition (DDA) analysis compared parental vs *MAEA*^*-/-*^ clones (Figure 1B, S2A, S2B, and Table S1). We also utilized a library free data-independent acquisition (DIA) method to quantitatively compare the ubiquitomes of parental, *MAEA*^*-/-*^cl8, and *resMAEA*^*-/-*^cl8 (Figure 1C, S2C, S2D, and Table S2). Out of 6266 (DIA) and 4046 (DDA) detected proteins we identified 15 (DDA) and 31 (DIA) as significantly MAEA-regulated proteins (5% p-value and >4-fold change cut-off), including subunits of the CTLH E3, in line with the previously described CTLH E3 autoubiquitylation (Figure S2G).^42,47^

Both DIA- and DDA-based proteomics experiments identified NMNAT1 as a potential substrate (Figure 1D). NMNAT1, a homohexamer, is one of three NMNAT isoforms catalyzing key steps in NAD^+^ biosynthesis.^49,50^ Immunoblot analysis of TUBE-enriched proteins showed NMNAT1 was precipitated from parental and *resMAEA*^*-/-*^, but not *MAEA*^*-/-*^ cells (Figure 1E). TUBE pulldown of NMNAT1 was abolished by treatment of cell lysates with the deubiquitylase OTUB1 (Figure S2H), indicating that NMNAT1’s presence in the MAEA-dependent ubiquitome depends on ubiquitin linkage.

### NMNAT1 ubiquitylation by WDR26-specific supramolecular CTLH E3 complex is independent of GID4

**Figure 2:**
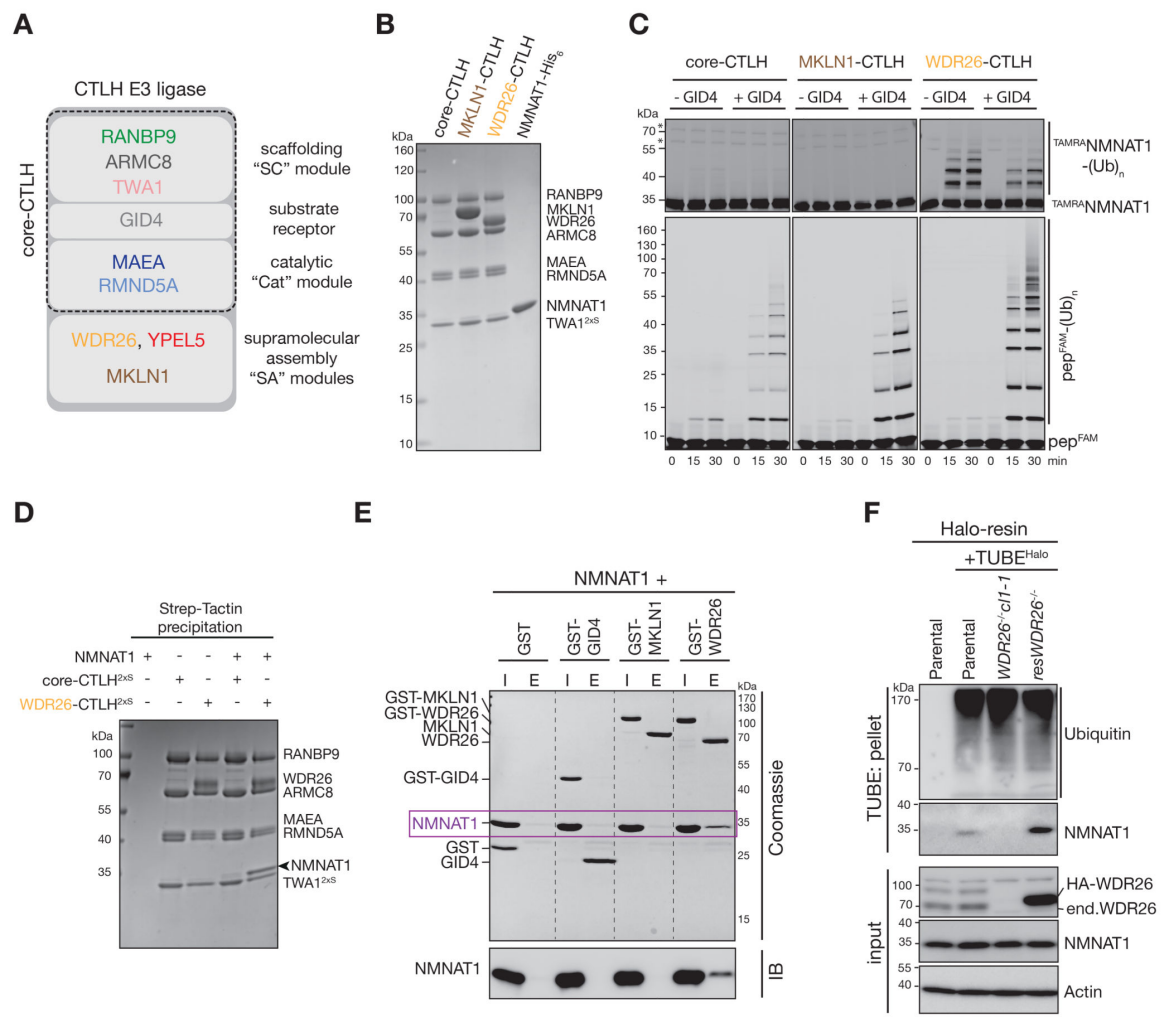
NMNAT1 ubiquitylation by WDR26-specific supramolecular CTLH E3 complex is independent of GID4 A) Cartoon with CTLH E3 complex subunits forming scaffold (SC), catalytic (Cat), and supramolecular assembly (SA) modules. SC and Cat modules assemble the core-CTLH E3. B) Coomassie-stained SDS-PAGE showing purified recombinant core-complex (RANBP9-TWA1^2xS^-ARMC8-RMND5A-MAEA), the supramolecular assemblies MKLN1-CTLH (RANBP9-TWA1^2xS^-ARMC8-RMND5A-MAEA-MKLN1) and WDR26-CTLH (RANBP9-TWA1^2xS^-ARMC8-RMND5A-MAEA-WDR26), and CTLH substrate NMNAT1. TWA1 is C-terminally tagged with 2xStrep. C) Fluorescent scans of SDS-PAGE gels presenting time course of *in vitro* ubiquitylation assay to test different CTLH E3 complex assemblies, in the absence or presence of GID4, for the ubiquitylation of N-terminally fluorescently tagged TAMRA-NMNAT1 (^TAMRA^NMNAT1) (top panels) and of fluorescently tagged model substrate peptide PGLW(X)_n_-23K-FAM (pep^FAM^) with lysine at position 23 (bottom panels). *, indicates unspecific products. D) Coomassie-stained SDS-PAGE showing Strep-Tactin precipitated samples of *in vitro* binding assay testing interaction between NMNAT1 and either core-CTLH or WDR26-CTLH E3 complexes comprising C-terminally 2xStrep-tagged TWA1 (TWA1^2xS^). E) For *in vitro* binding assay, NMNAT1 was incubated with individual GST-TEV-tagged CTLH subunits GID4, MKLN1, and WDR26 (I, input), followed by GST-affinity chromatography. GST-precipitates were treated with TEV to elute protein complexes (E, eluate) analyzed by Coomassie-stained SDS-PAGE and immunoblot (IB) analysis. F) TUBE^Halo^-captured proteins from indicated cell lines were precipitated with Halo-resin (TUBE: pellet) and analyzed by immunoblot analysis. Actin serves as protein input control.

We next asked whether NMNAT1 is a direct ubiquitylation substrate of the CTLH E3. We expressed and purified various recombinant human CTLH E3 complexes: the core CTLH E3 and its supramolecular assemblies formed with the two orthologs of yeast Gid7, WDR26 and MKLN1 (WDR26-CTLH E3 and MKLN1-CTLH E3, respectively, Figure 2A, 2B). All three complexes were active E3 ligases *in vitro*, because in the presence of GID4 they efficiently ubiquitylated a GID4-binding fluorescently-labeled model peptide substrate (pep^FAM^) (bottom panels, Figure 2C).^25^ However, ubiquitylation of fluorescently-labelled NMNAT1 (^TAMRA^NMNAT1) was not observed with either core-CTLH or MKLN1-CTLH E3. Rather, NMNAT1 was exclusively ubiquitylated by the WDR26-CTLH E3 complex, independent of GID4 (top panels, Figure 2C). Moreover, replacing wildtype ubiquitin with variants with either all lysines apart from K48 mutated to arginine, only K48 mutated to arginine, or all lysines mutated to arginine, suggested that multiple lysine sites on NMNAT1 are targeted by WDR26-CTLH E3 with a preference for K48-specific linkages as described recently for UBE2H/CTLH E3-catalyzed ubiquitylation (Figure S2I)^51^.

WDR26 dependency and GID4 independency of NMNAT1 ubiquitylation raised the question whether WDR26 is a NMNAT1 substrate receptor. To test this, we incubated purified NMNAT1 with 2xStrep-tagged core-CTLH or WDR26-CTLH E3, followed by capturing CTLH E3 complexes via Strep-Tactin precipitation. NMNAT1 was more efficiently co-precipitated with WDR26-CTLH E3 (Figure 2D). Moreover, upon incubating NMNAT1 with GST-tagged versions of individual CTLH subunits GID4, MKLN1, and WDR26, only WDR26 bound to and formed a stable complex with NMNAT1 (Figure 2E), suggesting a substrate receptor function of WDR26. In agreement, TUBE-enrichment from *WDR26*^*-/-*^ cells did not precipitate NMNAT1, whereas using *WDR26*^*-/-*^ cells re-expressing HA-tagged WDR26 (res*WDR26*^*-/-*^*)* led to efficient NMNAT1 precipitation (Figure 2F, S1B, S1E).

### Internal basic motif of NMNAT1 promotes binding and ubiquitylation by WDR26-CTLH E3

**Figure 3:**
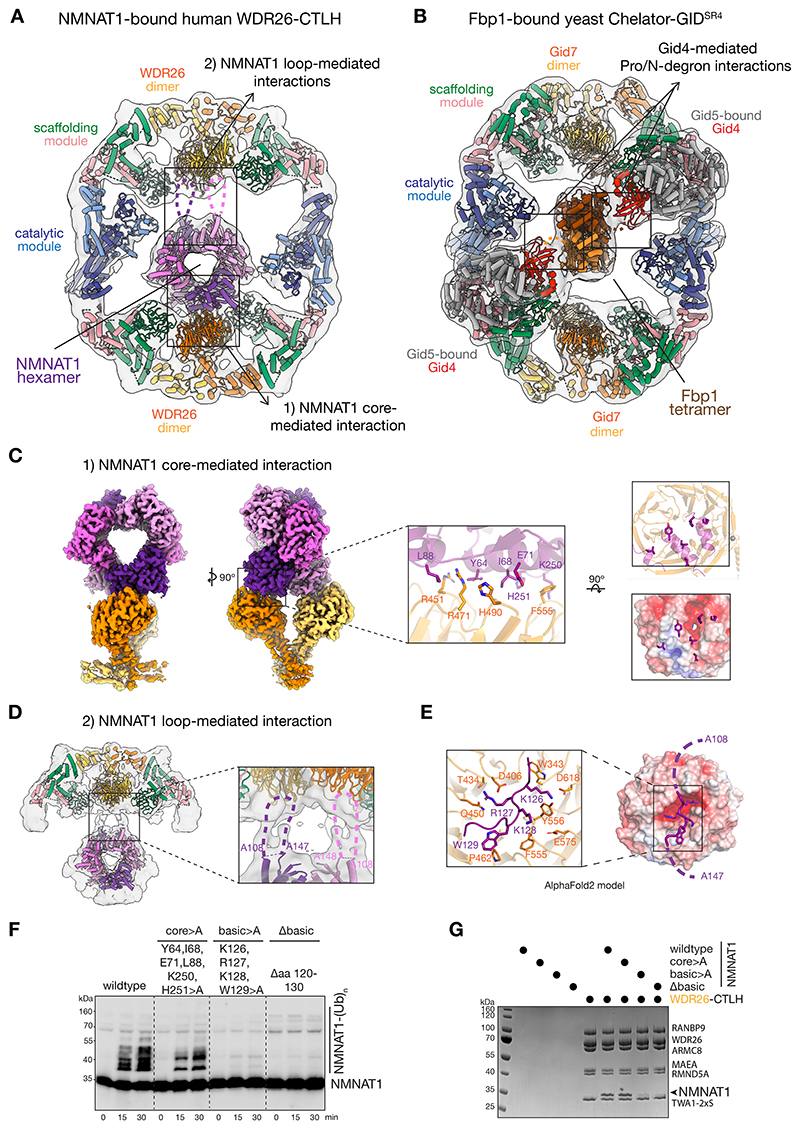
Internal basic motif of NMNAT1 promotes binding and ubiquitylation by WDR26-CTLH E3 A) 10-Å-resolution cryo-EM map of WDR26-CTLH E3 complex bound to NMNAT1 fit with a prior model of RANBP9-TWA1 (green and pink, respectively, extracted from PDB:7NSC), AlphaFold2 model of the catalytic module RMND5A-MAEA (dark blue and slate, respectively), WDR26 dimer (orange and yellow) as well as crystal structure of NMNAT1 hexamer (PDB: 1KQN, protomers colored in different shades of violet and pink) highlighting two modes of NMNAT1 interactions with the complex (indicated by black boxes). Dotted lines represent the flexible loops of two NMNAT1 protomers invisible in the crystal structure that extend towards WDR26 β-propellers at one side of the complex (visible in the low-resolution map at low contour). B) Cryo-EM map of Chelator GID^SR4^ bound to the tetrameric Fbp1 (EMD: 12577).^25^ C) Segmented 3.8-Å-resolution focused refined map of WDR26 dimer (excluding its CTLH-CRA^N^ domain) bound to the core domain of NMNAT1 sharpened with DeepEMhancer (top, proteins colored as in A). Close-ups of the corresponding model highlight NMNAT1’s core domain residues that interact with WDR26 residues (shown as sticks, center and left bottom) located in the largely hydrophobic portion of its β-propeller (represented as electrostatic potential surface, right bottom). D) 8.7-Å-resolution focused refined map of the NMNAT1^basic^-interacting part of WDR26-CTLH E3 complex, showing the low-resolution density for NMNAT1 loops (aa 109-146, dotted lines) extending towards WDR26 β-propellers. E) AlphaFold2 prediction model showing the basic motif of NMNAT1 loop (residues K126-W129) interacting with WDR26’s loops surrounding the central pore of its β-propeller (left: interacting residues from WDR26 and NMNAT1 shown as sticks; right: the negatively charged character of WDR26 central pore visible in its electrostatic potential surface representation). F) Immunoblot of *in vitro* assays testing WDR26-CTLH-mediated ubiquitylation of wildtype NMNAT1 (WT) and NMNAT1 variants mutating WDR26-interacting residues in the core domain (core>A) or NMNAT1 basic motif NMNAT1^basic^ (basic>A), and NMNAT1 with basic motif deletion (Δbasic). G) *In vitro* pull-down assay probing for the ability of wildtype (WT) and mutant NMNAT1 variants (tested in F) to bind WDR26-CTLH E3 complex (TWA1^2xS^-tagged) visualized by Coomassie-stained SDS-PAGE of Strep-Tactin-affinity elution fractions.

To visualize the GID4 independent NMNAT1 capture by the WDR26-CTLH E3, we performed cryo-EM (Figure S3A-C). All visualized NMNAT1-bound WDR26-CTLH E3 assemblies form giant hollow oval structures centrally encapsulating the hexameric NMNAT1 substrate (Figure 3A, S4), overall resembling the yeast Chelator-GID^SR4^ E3 encapsulating its tetrameric Fbp1 substrate (Figure 3B).^25^ However, unlike in the Chelator-GID^SR4^ E3 where dual substrate receptor Gid4 subunits bind N-terminal degrons of tetrameric Fbp1, the data suggested that the centrally facing β-propeller domains of the WDR26 dimers mediate NMNAT1 recruitment, thereby positioning multiple solvent-exposed NMNAT1 lysines in proximity to CTLH E3 catalytic domains (Figure 3A, S3E, and S3F).

The structural data showed multiple sites on NMNAT1 binding to WDR26. A 3.8-Å-resolution map obtained by focused refinement enabled placing atomic coordinates for the β-propeller domains of the WDR26 dimer on one side of the complex interacting with a pair of globular NMNAT1 core domains from neighboring protomers in the NMNAT1 hexamer (Figure 3C, S4, S5C-E). The NMNAT1 core domain does not fully cover the β-propeller surface but rather docks on its largely hydrophobic edge. Here, the loops within and between four WDR26 blades establish interactions with three NMNAT1 helices, which are distal to the NMNAT1 active sites (Figure 3C, S5D, and S5F). Nicotinamide-mononucleotide (NMN) is bound to NMNAT1 in the focused-refined map (see Methods). Notably, the prior structures of substrate, product, and a NAD^+^-mimetic drug-bound NMNAT1 are superimposable and can be docked onto the WDR26-bound complex, suggesting that recognition of NMNAT1 by WDR26-CTLH E3 would not be affected by occupancy of its active site (Figure S5F). Substituting the WDR26-interacting NMNAT1 core residues with alanines (core>A) only subtly impaired NMNAT1 ubiquitylation (Figure 3F) and binding to WDR26-CTLH E3 (Figure 3G), suggesting that other interactions anchor the complex.

Another NMNAT1-WDR26 interaction emerged from inspection of an 8.7-Å-resolution map focused over the opposite part of the complex. Here, long loops protrude from the globular cores of two adjacent NMNAT1 protomers towards WDR26’s β-propellers (Figure 3D). We presume that flexibility of the loops, and potential for each loop to contact each β-propeller, limits the conformational homogeneity of this region, and thus the resolution. Knowledge of these interacting regions based on the cryo-EM data guided the generation of a structure by AlphaFold2 for the NMNAT1 loops bound to WDR26. According to the model, part of the NMNAT1 loop spans across the central pore of WDR26 β-propeller interacting with its negatively charged entrance and surrounding elements (Figure 3E). Notably, a motif we term NMNAT1^basic^ due to its consisting of three basic residues (K126, R127 and K128), anchors WDR26. The complex is stabilized by numerous electrostatic interactions, and hydrophobic contacts mediated by W129 and the aliphatic portions of the basic sidechains (Figure 3E). In contrast to the NMNAT1 core domain mutant, alanine replacements for K126, R127, K128 and W129 in the NMNAT1^basic^ motif (basic>A), or deleting the basic motif (aa 120-130, Δbasic), impaired NMNAT1 ubiquitylation (Figure 3F) and binding to WDR26-CTLH (Figure 3G). Thus, the internal basic motif within NMNAT1’s disordered loop is critical for targeting NMNAT1 to the WDR26-CTLH E3.

### YPEL5 inhibits NMNAT1 ubiquitylation by WDR26-CTLH E3 and modulates cellular NMNAT1 turnover

**Figure 4:**
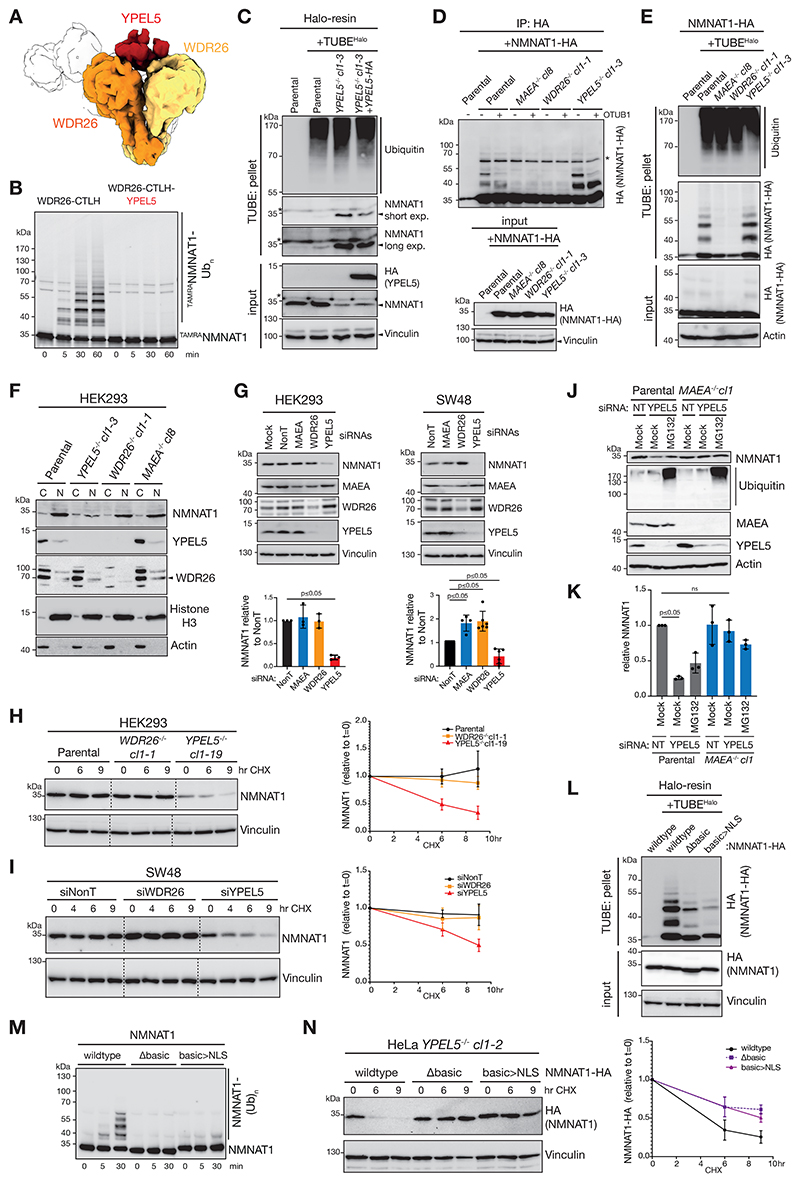
YPEL5 inhibits NMNAT1 ubiquitylation by WDR26-CTLH E3 and modulates cellular NMNAT1 turnover A) Previous 6.5-Å-resolution map of human RANBP9-TWA1-ARMC8-GID4-WDR26-YPEL5 subcomplex (EMBD: EMD-12545) highlighting YPEL5 (red) bound to the WDR26 dimer (orange and yellow). B) *In vitro* ubiquitylation assay testing effect of YPEL5 assembled into the WDR26-CTLH E3 complex on ubiquitylation of N-terminally fluorescently tagged TAMRA-NMNAT1 (^TAMRA^NMNAT1), visualized by a fluorescent scan of SDS-PAGE gel. C) TUBE^Halo^-captured proteins from HEK293 parental, *YPEL5*^*-/-*^, and YPEL5^*-/-*^ cells transiently expressing YPEL5-HA were precipitated with Halo-resin (TUBE: pellet) and analyzed by immunoblot. Vinculin serves as protein input control. D) To monitor NMNAT1 ubiquitylation *in vivo*, indicated HEK293 cell lines were transiently transfected with NMNAT1-HA. Cell lysates were split in half and either mock-treated or incubated with OTUB1 (-/+) prior to HA-immunoprecipitation (IP: HA) and subsequent immunoblot analysis (top panel). Input samples confirm equal NMNAT1-HA expression (bottom panel). Vinculin serves as input control. E) Indicated HEK293 cell lines were transiently transfected with NMNAT1-HA. TUBE^Halo^-captured proteins of cell lysates were precipitated with Halo-resin (TUBE: pellet) and analyzed by immunoblot. Input samples confirm equal NMNAT1-HA expression. Actin serves as input control. F) Lysates of HEK293 parental and CRISPR-Cas9 knockout cells were fractionated into cytosolic (C) and nuclear (N) fractions and analysed by immunoblot analysis. Histone H3 and Actin serve as nuclear and cytosolic makers, respectively. G) Indicated cell lines were mock-treated or subjected to siRNA targeting *MAEA, WDR26*, or *YPEL5*, and whole cell lysates analyzed by immunoblot (top panels). NonT, non-targeting siRNA control. Quantitation of NMNAT1 immunoblot signals normalized to Vinculin and relative to NonT control values (bottom panels). Graph shows results by means +/- SD of n=3. H) To assess the half-life of endogenous NMNAT1, indicated cell lines were treated with cycloheximide (CHX) for 0-9 hours, and cell lysates of each time point analyzed by immunoblot. Vinculin detection served as loading control. Quantitation of NMNAT1 immunoblot signals from H) normalized to Vinculin and relative to t=0 values (left panel). Graph plotting remaining NMNAT1 amounts (%), shows results by means +/- SD of n=5. I) Protein turnover of endogenous NMNAT1 was assessed in siWDR26*-* and siYPEL5-silenced SW48 cells by treated with cycloheximide (CHX) for 0-9 hours, and cell lysates of each time point analyzed by immunoblot. NonT, non-targeting siRNA control and Vinculin detection served as loading control. Quantitation of NMNAT1 immunoblot signals from I) normalized to Vinculin and relative to t=0 values. Graph plotting remaining NMNAT1 amounts (%), shows results by means +/- SD of n=2. J) HEK293 parental and *MAEA*^*-/-*^ knockout cells were subjected to non-target (NT) siNT and siYPEL5 followed by either mock or MG132 (10 µM) treatment overnight, and cell lysates were analyzed by immunoblot. K) Quantitation of NMNAT1 immunoblot signals from J) normalized to Actin and relative to mock-treated parental values. Graph shows results by means +/- SD of n=3. L) HEK293 cells were transiently transfected with NMNAT1-HA wildtype, NMNAT1-HA with basic motif deletion (NMNAT1^Δbasic^), and NMNAT1-HA with basic motif to NLS replacement (NMNAT1^basic>NLS^). TUBE^Halo^-captured proteins were precipitated with Halo-resin (TUBE: pellet) and analyzed by immunoblot (top panel). Input samples confirm equal expression of NMNAT1-HA and Vinculin serves as input control (bottom panel). M) Immunoblot of *in vitro* assays testing WDR26-CTLH-mediated ubiquitylation of wildtype NMNAT1 (WT), NMNAT1 with basic motif deletion (Δbasic), and NMNAT1 with the basic motif replaced with an NLS (basic>NLS). N) HeLa *YPEL5*^*-/-*^ cells transiently transfected with NMNAT1 variants as in L) and treated with cycloheximide (CHX) for 0-9 hours. Cell lysates of each time point were analyzed by immunoblot (right panel). Quantitation of NMNAT1 immunoblot signals normalized to Vinculin (serves as loading control) and relative to t=0 values (left panel). Graph plotting remaining NMNAT1-HA amounts shows results by means +/- SD of n=4.

Interestingly, we previously showed that the CTLH subunit YPEL5 associates with β-propeller regions of WDR26 dimers.^25^ Comparing the NMNAT1-bound WDR26-CTLH E3 to the previous map of a YPEL5-bound WDR26-CTLH subcomplex suggested potential overlap between the WDR26 binding sites of YPEL5 and the WDR26-interacting elements of NMNAT1 (Figure 4A). Indeed, the presence of YPEL5 in a WDR26-CTLH E3 complex abolished ubiquitylation of NMNAT1 *in vitro* (Figure 4B). Consistent with an inhibitory function, TUBE-enrichment from YPEL5-deficient cells showed measurable enhancement of NMNAT1 precipitation compared to parental cells, potentially due to elevated NMNAT1 ubiquitylation (Figure 4C). To further assess NMNAT1 ubiquitylation *in vivo*, C-terminally HA-tagged NMNAT1 (NMNAT1-HA) was expressed in parental, *MAEA*^*-/-*^, *WDR26*^*-/-*^, and *YPEL5*^*-/-*^ cells. NMNAT1-HA immunoprecipitation revealed NMNAT1 species modified with several ubiquitin moieties which were - consistent with our *in vitro* evidence for K48-linked ubiquitylation (Figure S2I) - reduced upon K48-specific OTUB1 treatment, and importantly, undetectable in *MAEA*^*-/-*^ and *WDR26*^*-/-*^ cells (Figure 4D). Notably, detected NMNAT1 ubiquitylation is elevated in *YPEL5*^-/-^ cells, which is supported by complementary analysis using TUBE-enrichment of NMNAT1-HA (Figure 4E).

We further queried the inhibitory function of YPEL5 in cells on NMNAT1 cellular levels. NMNAT1 is best known to function in the nucleus.^52,53^ Although WDR26 and YPEL5 were found to localize primarily in the cytoplasm and to a far lesser extent in the nucleus,^46,54^ the nuclear CTLH E3 assembly was shown to be substantially disrupted upon loss of WDR26.^46^ Interestingly, subcellular fractionation revealed that the amount of NMNAT1 is reduced in the nuclear fraction of *YPEL5*^*-/-*^, but unexpectedly not affected in *MAEA*^*-/-*^ and *WDR26*^*-/-*^ HEK293 cells (Figure 4F, S6A). We speculated that WDR26-CTLH E3 might be largely occupied by YPEL5 in HEK293 cells, and hence depletion of WDR26 is not resulting in increased NMNAT1 amounts. To address potential cell type-specific regulation, we expanded our studies using a selection of cell lines that have different WDR26 and YPEL5 RNA expression profiles (https://www.proteinatlas.org/humanproteome/cell+line). Indeed, immunoblot analysis revealed differences of YPEL5, WDR26, and NMNAT1 protein amounts (Figure S6B). siRNA-mediated silencing of *YPEL5* significantly reduced NMNAT1 amounts in all tested cell lines (Figure 4G and S6C). In contrast, NMNAT1 significantly accumulated only in SW48 cells upon siMAEA or siWDR26 silencing, suggesting cell type-specific regulation of NMNAT1 amounts by YPEL5-WDR26-CTLH E3. Moreover, subcellular fractionation of *YPEL5* CRISPR-Cas9-edited (*YPEL5sg*) SW48 cells revealed a measurable reduction, whereas *WDR26sg* cells an accumulation of nuclear NMNAT1 (Figure S6D). We also noted that YPEL5 amounts were largely reduced in all tested WDR26-deficient cell lines, suggesting that in the absence of the binding partner WDR26, YPEL5 becomes unstable (Figure 4F, 4G, S6A, S6C, and S6D). Next, to test whether YPEL5’s and WDR26-CTLH E3’s effect on NMNAT1 amounts is due to altered protein stability, HEK293 parental and knockout cells were treated with the translation inhibitor cycloheximide (CHX) (Figure 4H). Overall, after different treatment intervals only *YPEL5*^*-/-*^ cells showed significant destabilization of NMNAT1 amounts, whereas parental and *WDR26*^*-/-*^ cells did not show a detectable turnover of NMNAT1 after 9 hr. Similar observations were made using siYPEL5 and siWDR26-silenced SW48 cells (Figure 4I).

To further investigate the NMNAT1 degradation mechanism, HEK293 parental and *YPEL5*^*-/-*^ cells were either mock treated or exposed to the proteasomal inhibitor MG132. In agreement with our concept of enhanced proteasomal degradation of NMNAT1 in *YPEL5*^*-/-*^ cells, MG132 treatment stabilized NMNAT1 to levels detected in parental cells (Figure S6E and S6F). Next, we assessed whether destabilization of NMNAT1 in *YPEL5*-deficient cells is dependent on the catalytic activity of the CTLH E3 complex. YPEL5 was depleted in HEK293 parental and CTLH E3 inactive *MAEA*^*-/-*^ cells by siRNA silencing followed by mock or MG132 treatment. NMNAT1 levels were significantly reduced in *YPEL5*-silenced parental cells but stabilized upon MG132 treatment (Figure 4J, 4K, S6E, and S6F). In contrast, *YPEL5* silencing in *MAEA*^*-/-*^ cells had no significant effect on NMNAT1 amount, suggesting that the absence of YPEL5 promotes MAEA-mediated proteasomal degradation of NMNAT1.

Next, we examined whether the basic motif of NMNAT1 is required for *in vivo* ubiquitylation of NMNAT1. However, the basic motif is also part of NMNAT1’s nuclear localization signal (NLS) (Figure S5A).^55^ Indeed, transiently transfected basic motif-deleted NMNAT1 (NMNAT1^Δbasic^) accumulates nearly exclusively in the cytosolic fraction and hence might escape nuclear WDR26-CTLH E3 targeting (Figure S6G). However, replacing the basic motif of NMNAT1 with a conventional nuclear localization signal (NLS) sequence (NMNAT1^basic>NLS^) maintained its predominant nuclear localization (Figure S6G), but importantly showed defective ubiquitylation *in vivo* (Figure 4L) and *in vitro* (Figure 4M). Moreover, NMNAT1^basic>NLS^ has a significantly reduced protein turnover compared to wildtype NMNAT1 in *YPEL5*^*-/-*^ cells (Figure 4N). Thus, the data suggests that the NMNAT1 basic motif is bifunctional encoding an NLS and a WDR26-selective internal degron.

### YPEL5 and WDR26-CTLH E3 modulate NMNAT1-mediated prodrug metabolism

**Figure 5:**
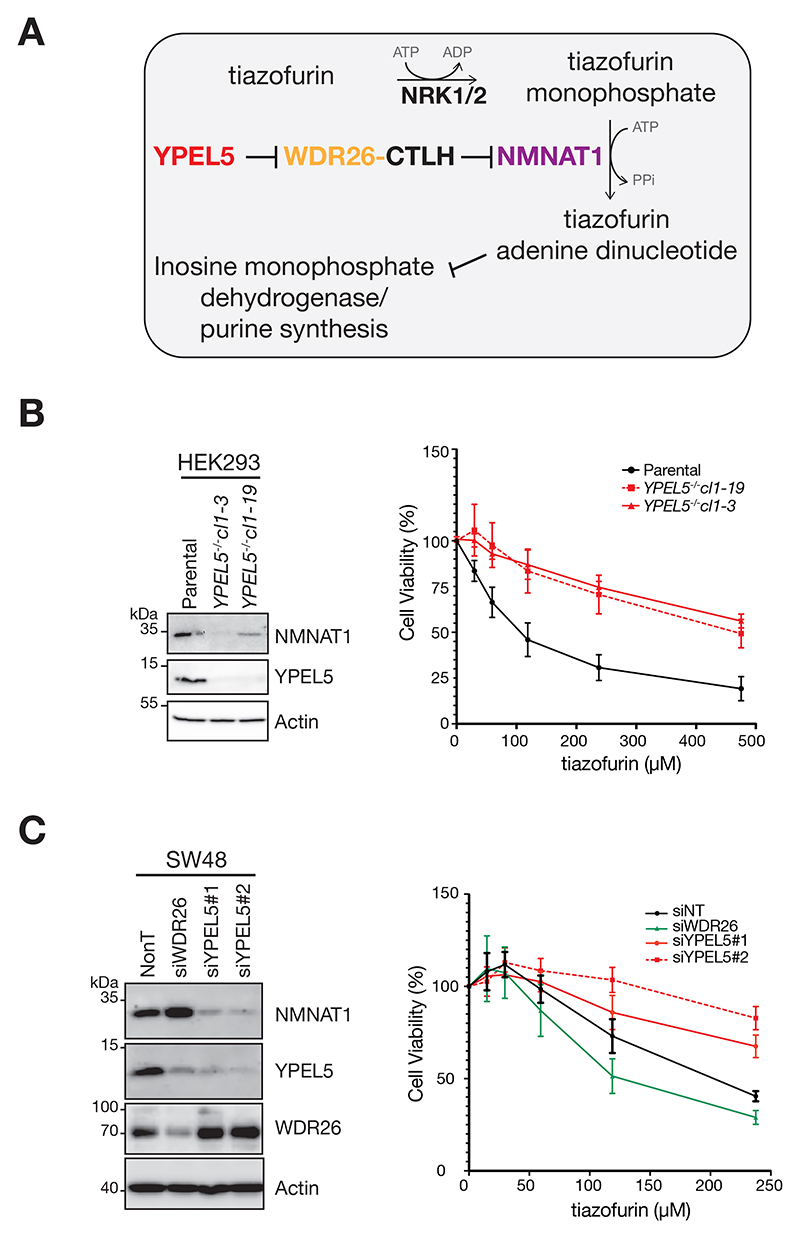
YPEL5 and WDR26-CTLH E3 modulate NMNAT1-mediated prodrug metabolism A) Reaction cascade of the metabolic conversion of prodrug tiazofurin to bioactive tiazofurin adenine dinucleotide (TAD). TAD inhibits inosine monophosphate dehydrogenase attenuating purine synthesis. B) HEK293 *YPEL5*^*-/-*^ cells (clones 1-19 and 1-3) were treated with tiazofurin (0-475 µM), and cell viability assessed after 96 hours. *YPEL5*^*-/-*^ lines were confirmed by immunoblot analysis (left). Graph of cell viability (right) shows results by means +/- SD of n=3. C) SW48 cells were subjected to non-target siNonT, siWDR26, and siYPEL5 followed by treatment with tiazofurin (0-240 µM), and cell viability assessed after 96 hours. WDR26 and YPEL5 depletion was confirmed by immunoblot analysis (left). Graph of cell viability (right) shows results by means +/- SD of n=3.

We next made use of the anti-cancer agent tiazofurin to probe the functional link between YPEL5 and NMNAT1 stability. Tiazofurin is a prodrug metabolized in a two-step reaction utilizing nicotinamide riboside kinase 1/2 (NRK1/2) and NMNAT1 activities to generate the toxic bioactive NAD^+^-mimetic thiazol-4-carboxamide-adenine dinucleotide (TAD).^56,57^ TAD acts as a non-competitive inhibitor of inosine-monophosphate-dehydrogenase IMPDH and showed high selectivity against human IMPDH2 versus other cellular dehydrogenases.^57^ IMPDH2 is the rate limiting enzyme in GTP synthesis, hence tiazofurin treatment results in cell growth inhibition (Figure 5A). As tiazofurin’s bioactivity depends on NMNAT1, tiazofurin efficacy can readout changes in NMNAT1 function. As proof of concept, *siNMNAT1*-silenced HEK293 cells were treated with increased concentration of tiazofurin and cell growth was assessed after 96 hours. Indeed, *siNMNAT1* cells were significantly more resistant to tiazofurin compared to non-target (NonT) control cells (Figure S6H). Importantly, resistance was observed in siYPEL5-depleted cells correlating with reduced NMNAT1 amounts. Moreover, the analysis of HEK293 *YPEL5*^*-/-*^ cells also showed tolerance to increased tiazofurin concentration in comparison to parental cells (Figure 5B). We extended the analysis to SW48 cells revealing that siYPEL5-silencing caused reduced, whereas siWDR26-silencing increased tiazofurin cytotoxicity, correlating with reduced and elevated NMNAT1 amounts, respectively (Figure 5C). Cumulatively, the data suggest that YPEL5 modulates WDR26-CTLH E3 activity controlling NMNAT1-mediated prodrug metabolism.

### N-terminus of YPEL5 mimics the internal NMNAT1^basic^ motif

**Figure 6:**
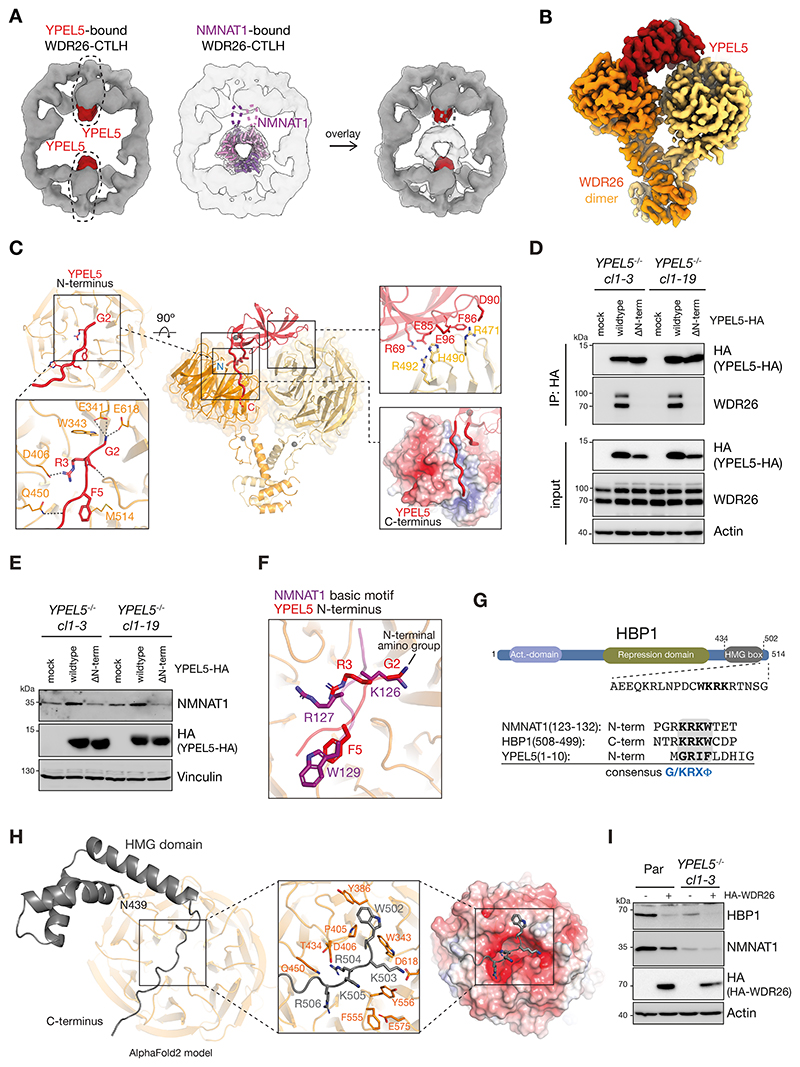
N-terminus of YPEL5 mimics NMNAT1^basic^ degron A) Comparison of low-resolution cryo-EM reconstructions of YPEL5-bound (left, YPEL5 shown in red) and NMNAT1-bound (middle, fit with crystal structure of NMNAT1 as in figure 3A, loops highlighted by dotted lines) WDR26-CTLH complex. The overlay of the two maps (right) reveals overlap between YPEL5 and both NMNAT1-binding sites in the opposing WDR26 dimers. B) Segmented 3.2-Å-resolution focused refined map of YPEL5-bound WDR26 dimer (excluding its CTLH-CRA^N^ domain). C) Overview of three WDR26-binding elements of YPEL5: 1) residues at the extreme YPEL5 N terminus interacting with the loops surrounding the central pore of WDR26 β-propeller (left); 2) YPEL5 surface encompassing strands and connecting loops from its central β-sheet docked at the side of the second WDR26 β-propeller (right, top); 3) the peptide-like YPEL5 C-terminus interacting with the largely basic groove formed by the blades of WDR26 β-propeller that also binds YPEL5 N terminus (right, bottom). D) HEK293 *YPEL5*^*-/-*^ cells (clones cl1-3 and cl1-19) were mock-transfected or transfected with either C-terminally HA-tagged wildtype YPEL5 or YPEL5 with an N-terminal deletion (ΔN-term). HA-immunoprecipitates were analyzed by immunoblot analysis. E) HEK293 *YPEL5*^*-/-*^ cells (clones cl1-3 and cl1-19) were transfected as in D) and whole cell lysates analyzed by immunoblot analysis. Vinculin detection served as loading control. F) Overlay of NMNAT1^basic^ and YPEL5 N-terminus bound to WDR26 β-propeller (from AlphaFold2 prediction and cryo-EM structure, respectively). G) Domain structure of HBP1 indicating activation and repression domains and the High-Mobility-Group (HMG) box (top). HBP1 extreme C-terminal amino acid sequence (aa 502-514) with potential basic motif degron sequence (bold and underlined) aligning with NMNAT1 and YPEL5 binding motifs revealing a consensus sequence (ϕ represents amino acids with a bulky hydrophobic residue including W, Y, F). H) AlphaFold2 model of HBP1 C-terminus (aa 439-510) binding to WDR26 β-propeller. Close-up shows the disordered region of HBP1 (downstream of its HMG box) interacting with the central negatively-charged pocket of WDR26 β-propeller (bottom, left) mediated by the WDR26-binding consensus basic motif. I) HEK293 parental and *YPEL5*^*-/-*^ knockout cells were either mock-treated or transfected with C-terminal HA-tagged WDR26 and cell lysates analyzed by immunoblot.

Cryo-EM maps revealed how YPEL5 inhibits substrate targeting: there is structural overlap between YPEL5 and NMNAT1 binding sites on WDR26-CTLH (Figure 6A, S7). Molecular details of YPEL5 binding to WDR26 were revealed by a 3.2-Å-resolution focused refined map (Figure 6B). YPEL5 simultaneously engages both WDR26 protomers in an asymmetric manner via three structural features (Figure 6C): (1) YPEL5’s N-terminus stretching across the central pore of one WDR26 β-propeller (left zoom-in Figure 6C); (2) its C-terminus whose trajectory complements a distinctive groove at the side of the same β-propeller (right bottom zoom-in Figure 6C); and (3) the YPEL5 folded domain, which consists of two β-sheets packing against each other, binding at the edge of the second β-propeller in the WDR26 dimer (right top zoom-in Figure 6C). To further investigate the importance of YPEL5’s N-terminus for binding WDR26, C-terminally HA-tagged YPEL5 wildtype or YPEL5 with an N-terminal deletion (YPEL5^ΔN-term^) were expressed in *YPEL5*^*-/-*^ cells. Whereas YPEL5-HA efficiently co-precipitate WDR26, binding to WDR26 was completely abolished in the absence of YPEL5’s N-terminus (Figure 6D). Moreover, in contrast to wildtype YPEL5-HA, expression of YPEL5^ΔN-term^ was unable to stabilize NMNAT1 amounts (Figure 6E).

Strikingly, the overlay of NMNAT1^basic^ motif and YPEL5 N-terminus bound to WDR26 β-propeller revealed similar elements mediating the interactions (Figure 6F). From YPEL5, the N-amino group of G2 (exposed upon cleavage of initiator M1) and the side chains of R3 and F5 are anchored through a similar constellation of electrostatic and hydrophobic contacts as the γ-amino group of K126 side chain, R127 and W129 from NMNAT1^basic^ (Figure 6F). Comparing the NMNAT1^basic^ and YPEL5 N-terminal sequences reveals a consensus G/K-R-X-ϕ basic motif (ϕ represents amino acids with a bulky hydrophobic residue including W, Y, F) that might be selectively recognized by WDR26 (Figure 6G).

Based on identification of this motif, we reevaluated the results from our TUBE/MS experiments identifying MAEA-dependent ubiquitylation targets (Figure 1B, C, and S2G). In addition to NMNAT1, our experiment identified HBP1, which was previously reported as a WDR26-dependent substrate.^41,42^ Inspection of the HBP1 sequence revealed an inverted (C- to N-terminus) arrangement of the motif, K-R-K-W (aa 505-502), downstream of the High-Mobility-Group (HMG) domain of HBP1 (Figure 6G). To test potential for this motif to engage the same binding interface as NMNAT1, we obtained an AlphaFold2-predicted model of this region of HBP1 bound to WDR26 (Figure 6H). The model places the C-terminus of HBP1 across the WDR26 β-propeller with the consensus motif in the center establishing similar interactions as NMNAT1^basic^ and YPEL5. Indeed, similar to NMNAT1, HBP1 abundance was reduced in *YPEL5-*deficient cells, and WDR26-HA overexpression reduced the level of HBP1 in parental and *YPEL5*^*-/-*^ cells (Figure 6I). Taken together, we propose that YPEL5 inhibits WDR26’s substrate receptor function by mimicking internal basic substrate degrons, thereby preventing substrate ubiquitin targeting.

## Discussion

Numerous E3 ligases are multi-subunit complexes with interchangeable substrate receptors that engage and enable efficient ubiquitylation of diverse substrates. Such a mode of substrate recognition is structurally well-characterized for yeast GID E3 complexes with interchangeable Gid4-family receptors recognizing N-degrons. The human GID4 ortholog mediates similar interactions. Nonetheless, several prior studies have shown that the human CTLH E3 forms related but diverse assemblies.^25,41,43,44,46,58^ Although WDR26 had been proposed as a substrate receptor, how this subunit - which is intrinsic to a supramolecular WDR26-CTLH E3 complex - could mediate substrate binding remained unknown. Here, our interdisciplinary approach combining ubiquitin-affinity proteomics from cells engineered to lack CTLH E3 ligase activity, biochemistry, and structural biology identified NMNAT1 as a WDR26-CTLH E3 substrate, defined the degron recognized by WDR26, and degron mimicry by YPEL5 preventing substrate ubiquitylation.

Within the supramolecular WDR26-CTLH E3 assembly, the WD40-repeat β-propeller domains from two WDR26 dimers are exposed and provide multiple binding surfaces for NMNAT1. These interactions differ from substrate recruitment to Gid4-family receptors, which belong to the calycin class of β-barrel proteins. Prior structures showed that the β-barrels of Gid4-family members form a narrow funnel that is specifically tailored to selectively capture substrate N-termini and downstream residues.^21,25,32–34,59^ However, the WDR26 β-propeller domain can capture its cognate basic motif located either at a protein terminus, as shown here for YPEL5, or internally within a protein loop as shown for NMNAT1 (Figure 6F). The dimeric arrangement of WDR26 seems to impart specificity through its capacity to form avid interactions either with additional portions of a monomeric partner like YPEL5, or the oligomeric target NMNAT1. Thus, WDR26 extends the substrate range of the CTLH E3 beyond those with N-degrons. We speculate that the other Gid7 ortholog in higher eukaryotes, MKLN1, could recognize substrates in a manner conceptually analogous to WDR26.^39,40^

The common structural features of WDR26 interactions with the NMNAT1’s basic motif and YPEL5’s N-terminus guided the identification of a consensus motif, K-R-X-ϕ, as a signature of WDR26-selective substrates. Importantly, this motif is not found in the structurally related non-substrates NMNAT2 and NMNAT3 (Figure S5A), but is shared by the previously-described WDR26-dependent CTLH substrate HBP1,^41,42^ which was also identified in our proteomics experiments (Figure 1C). Indeed, an AlphFold2 model shows HBP1 binding the WDR26 β-propeller similarly to NMNAT1 and YPEL5. Although HBP1 differs from NMNAT1 in that it is not known to form dimers or oligomeric assemblies, HBP1 does function in chromatin-bound macromolecular complexes with several binding partners.^60,61^ Future studies illuminating HBP1 binding to the WDR26-CTLH complex will be required to determine if the E3 recognizes HBP1 as a monomer much like its binding to YPEL5, or if HBP1-binding partners contribute to complex formation much like for the NMNAT1 self-assembly. Furthermore, some other CTLH E3 substrates selectively recruited by WDR26 - potentially specific to particular cell-types - are likely to exist and will facilitate a better understanding of WDR26-specific biological processes and pathologies.^29,54,62,63^

**Figure 7:**
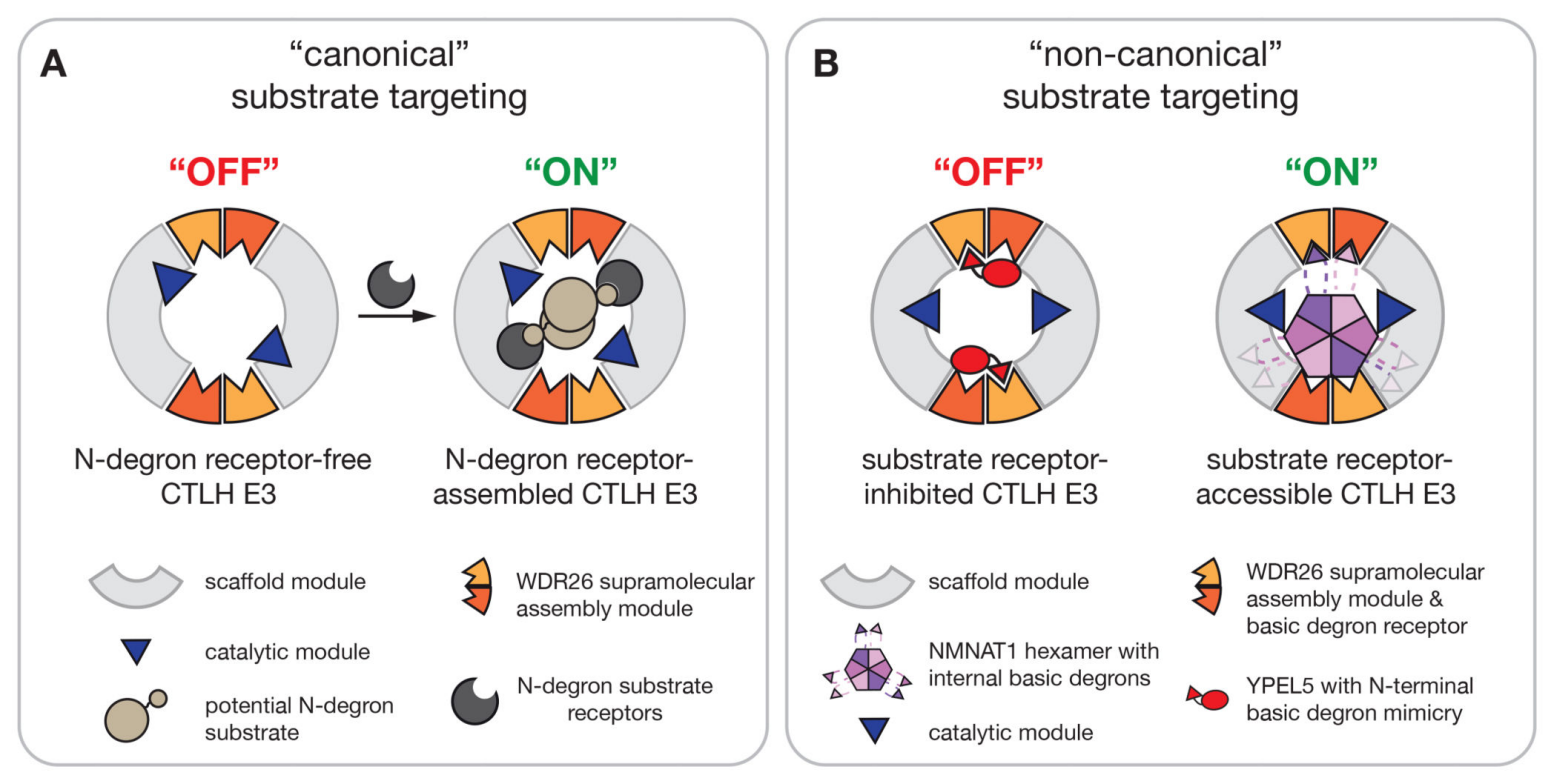
Model of canonical versus non-canonical regulation of substrate binding by human WDR26-CTLH E3 ligase complexes A) Canonical substrate binding and regulation: N-degron substrates are recruited by removable/interchangeable N-degron receptors, whereas WDR26 dimers function as supramolecular assembly modules. B) Non-canonical substrate binding and regulation: Inherent WDR26 dimers function as an assembly module and as a substrate receptor module to bind internal basic degrons of substrate NMNAT1. YPEL5’s N-terminus mimics basic degrons of substrates, thereby inhibiting WDR26 substrate receptor function.

An emerging theme of E3 ligase complex regulation is inhibition of substrate binding by mimetic cellular factors. For example, inhibition of degron binding was found in regulating interchangeable substrate receptors of CRLs, such as the C-degron KLHDC2 receptor of the cullin-2 RING ligase (CRL2). There, the C-terminus of one KLHDC2 mimics the C-degron and engages the substrate binding domain of another KLHDC2 promoting an autoinhibitory self-assembly that prevents non-substrate binding.^64^ Another inhibitory concept was proposed for members of the BEX pseudosubstrate inhibitor of the reductive stress ligase CUL2-FEM1B.^65^ BEX mimics the zinc-dependent substrate interaction of the mitochondrial gate keeper FNIP1 in its reduced state, which prevents FNIP1 degradation to protect cells from ROS accumulation. We discovered a mimicry-type mechanism also regulates CTLH E3 substrate targeting. Our structural analysis revealed multivalent binding of a single YPEL5 to both WDR26 β-propeller domains in a WDR26 dimer. YPEL5’s N-terminus presents a sequence that mimics NMNAT1’s internal basic motif that together with YPEL5’s C-terminus captures one WDR26 protomer. Meanwhile, YPEL5’s globular domain binds the adjacent WDR26 protomer. As such, YPEL5 modulates WDR26’s accessibility to degrons from *bona fide* substrates (Figure 7). Interestingly, a comparable inhibitory mechanism was recently described to control the activity of the BIRC6 E3 ligase. BIRC6 is competitively inhibited by a multivalently bound SMAC dimer that occludes substrate binding sites. Notably, SMAC’s N-terminus mimics a substrate degron anchored to the BIR domain.^66–68^

YPEL5’s role is further supported by our cellular studies, showing that NMNAT1 amounts are reduced, due to proteasome-dependent destabilization, in YPEL5-deficient cells. NMNAT1 is amongst three human NMNAT proteins that produce NAD^+^. NMNAT1 is primarily thought to determine NAD^+^ levels in the nucleus, to fuel regulatory pathways that depend on numerous enzymes consuming this and related cofactors.^52,53^ NMNAT1 is required for differentiation and development of some specialized cell types, including adipocytes, which rely on NAD^+^-consuming enzymes for gene regulation, and photoreceptors, which depend on NAD^+^ to prevent apoptosis.^69,70^ However, the NMNAT1 gene is homozygously deleted in certain cancers; notably, glioma cell lines lacking NMNAT1 depend on NMNAT2 for viability.^71^ Despite the complexity of NAD^+^/NADH homeostasis, NMNAT1 is required for cellular activation of the NAD^+^-mimetic drug tiazofurin. Indeed, we found that YPEL5 deficiency mitigates tiazofurin cytotoxicity, indicating that cellular NMNAT1 activity is subject to modulation by YPEL5. All cell types tested in this study showed reduced NMNAT1 amounts upon depletion of YPEL5, one of which, the colon rectal cancer cell line SW48, also showed a corresponding increase in NMNAT1 amounts - and increased susceptibility to tiazofurin - upon WDR26 depletion (Figures 4G, 5C, S6C). We note that our study complements work by the Luis Parada laboratory describing similar effects of YPEL5 and WDR26 deletion in mouse cells on NMNAT1 amounts, and on modulating the efficacy of a candidate prodrug in the killing of specific types of cancer cells (personal communication). Thus, we anticipate that YPEL5-WDR26-CTLH E3 ubiquitylation will emerge as critical mechanism with wide-ranging implications in NMNAT1-dependent prodrug metabolism.

### Limitations of the study

Our structural and cellular studies suggest NMNAT1 ubiquitylation by the WDR26-CTLH E3, and inhibition by YPEL5. Important cellular consequences are indicated by MAEA’s strong co-dependency with NMNAT1 reported in DepMap (https://depmap.org/portal), but physiological roles are likely cell and/or organ type specific.

We speculate that the stoichiometries of WDR26 and/or YPEL5 interactions vary between cell lines. If WDR26-CTLH E3 was highly occupied by YPEL5 in most cell lines we studied, this could explain why loss of WDR26 would not substantially impact amounts of NMNAT1. Given that YPEL5 appears to modulate the amount of nuclear NMNAT1, this study did not further address whether there is a signal that triggers NMNAT1 recognition by the WDR26-CTLH E3. Potential types of signals may be gleaned from existing paradigms. For example, a recent study showed that the cytoplasmic SIFI E3 ligase complex recognizes degrons corresponding to mitochondrial targeting sequences, such that the E3 targets mislocalized mitochondrial proteins.^72^ It is tempting to speculate that there could be a conceptually related regulation mediated by the WDR26-CTLH E3 and its capture of NLS sequences. Moreover, we provide only limited insights into whether and how YPEL5’s inhibition is relieved to allow ubiquitin-targeting of NMNAT1 and other potential substrates by WDR26-CTLH E3. It is possible that certain changes in cellular conditions could effect YPEL5/WDR26 interaction, nuclear localization of the WDR26-CTLH E3 ligase,^46^ or elicit nuclear destruction of YPEL5. Interestingly, we observed increased YPEL5 abundance in MAEA-deficient cells and upon proteasomal inhibition (Figure 4F, S6A, and S6E), raising the possibility that WDR26-CTLH E3 complex might control YPEL5 amounts by proteasomal degradation. Finally, a recent study has pointed toward genetic and epigenetic regulation of *YPEL5* expression.^73^

## Star Methods

### Resource Availability

**Table:** Key Resources Table

REAGENT or RESOURCE	SOURCE	IDENTIFIER
**Antibodies**		
Mouse monoclonal anti-NMNAT-1 (B-7) antibody	Santa Cruz	Cat#sc-271557; RRID: AB_10647226
Sheep polyclonal anti-MAEA antibody	R&D Systems	Cat# AF7288-SP, RRID: AB_10971438
Rabbit polyclonal anti-RANBP9 antibody	Abnova	Cat# PAB16671; RRID: AB_10677213
Mouse monoclonal anti-ARMC8 antibody	Santa Cruz	Cat# sc-365307; RRID: AB_10850172
Rabbit polyclonal anti-WDR26 antibody	Abcam	Cat# ab85962; RRID: AB_1925564
Mouse monoclonal anti-MKLN1 antibody	Santa Cruz	Cat# sc-398956; RRID: AB_2737249
Rabbit polyclonal anti-YPEL5 antibody	Thermo Fisher Scientific	Cat# PA5-26957; RRID: AB_2544457
Mouse monoclonal Anti-Ubiquitinylated proteins clone FK2 antibody	Millipore	Cat# 04-263, RRID: AB_612093
Rabbit monoclonal β-Actin(13E5) antibody	Cell Signaling Technology	Cat# 4970, RRID: AB_2223172
Rabbit monoclonal Vinculin antibody [EPR8185]	Abcam	Cat# ab129002, RRID: AB_11144129
Rabbit polyclonal HBP1 antibody	Abcam	Cat# ab83402, RRID: AB_1860581
Mouse monoclonal ANTI-FLAG M2 antibody	Sigma-Aldrich	Cat# F1804, RRID: AB_262044
Mouse monoclonal HA-Tag (6E2) antibody	Cell Signaling Technology	Cat# 2367, RRID: AB_10691311
HaloLink Resin	Promega	Cat# G1912
His-Select Nickel affinity gel	Sigma Aldrich	Cat# P6611
Glutathione Sepharose 4B	GE Healthcare	Cat# 17075605
Strep-Tactin Sepharose High Performance resin	Cytiva	Cat# 28935599
		
**Bacterial and Virus Strains**		
*E. coli* BL21 RIL (DE3)	MPIB	N/A
*E. coli* DH5α	MPIB	N/A
		
**Chemicals, Peptides, and Recombinant Proteins**		
Complete EDTA free protease inhibitor cocktail	Roche	Cat# 05056489001
Aprotinin from bovine lung	Sigma Aldrich	Cat# A1153-10MG
Leupeptin	Sigma Aldrich	Cat# L2884-250MG
HALT protease and phosphatase inhibitor cocktail	Thermo Fisher	Cat# 78441
Benzonase Nuclease	Millipore	Cat# E1014
MG132	Sigma Aldrich	Cat# M8699
Tiazofurin	BLD Pharm	Cat# BD154096
Peptide: PGLWRSPRRDSTEGFTGRGWSGRG WSKGGK-FAM	Sherpa et al.^25^	N/A
		
**Critical Commercial Assays**		
CellTiter-Glo Luminescent Cell Viability Assay	Promega	Cat# G7571
Micro BCA Protein Assay Kit	Thermo Fisher Scientific	Cat# 23235
ECL Western Lightning Plus	Perkin Elmer	Cat# NEL104001EA
		
**Deposited Data**		
NMNAT1-bound WDR26-CTLH E3 assembly I, class 1	This study	EMD-18174
NMNAT1-bound WDR26-CTLH E3 assembly I, class 2	This study	EMD-18175
NMNAT1-bound WDR26-CTLH E3 assembly II, class 1	This study	EMD-18176
NMNAT1-bound WDR26-CTLH E3 assembly II, class 2	This study	EMD-18177
NMNAT1-bound WDR26-CTLH E3 assembly II, class 3	This study	EMD-18178
RANBP9-TWA1-WDR26 module binding core NMNAT1	This study	EMD-18172
RANBP9-TWA1-WDR26 module binding NMNAT1 loops	This study	EMD-18173
Structure of NMNAT1-bound WDR26 dimer	This study	EMD-18345 PDB 8QE8
YPEL5-bound WDR26-CTLH E3, assembly I	This study	EMD-18170
YPEL5-bound WDR26-CTLH E3, assembly II	This study	EMD-18171
Structure of YPEL5-bound WDR26 dimer	This study	EMD-18316 PDB 8QBN
Raw image data, Mendeley data	This study	DOI: 10.17632/cnv6tn2dzf.1
Proteomics data	This study	Table S1, S2, PRIDE: PXD044126
		
**Experimental Models: Cell Lines**		
Flp-In-T-Rex-HEK293	ATCC	ATCC#CRL-1573; RRID: CVCL_U427
Flp-In-T-Rex-HeLa	Gift from Christian Behrends (Ludwig-Maximilians-University, Germany	N/A
U2OS	ATCC	ATCC# HTB-96; RRID: CVCL_0042
SH-SY5Y	ATCC	ATCC# CRL-2266; RRID: CVCL_0019
SW48	ATCC	ATCC# CCL-231; RRID: CVCL_1724
Sf9 Insect cells	Thermo Fisher Scientific	Cat# 11496015
High Five Insect cells	Thermo Fisher Scientific	Cat# B85502
		
**Oligonucleotides**		
ON-TARGETplus Non-targeting siRNA	Dharmacon Reagents	Cat# D-001810-10
MISSION esiRNA targeting human MAEA	Sigma Aldrich	Cat# EHU065961
MISSION esiRNA targeting human WDR26	Sigma Aldrich	Cat# EHU150671
MISSION esiRNA targeting human YPEL5	Sigma Aldrich	Cat# EHU130581
MISSION esiRNA targeting human NMNAT1	Sigma Aldrich	Cat# EHU016171
ON-TARGETplus Human YPEL5 siRNA Smart pool	Dharmacon Reagents	Cat# L-020317-01
		
**Recombinant DNA**		
pcDNA-5/FRT	Thermo Fisher Scientific	Cat# V601020
pcDNA-3.1/Hygro	Thermo Fisher Scientific	Cat# V87020
pcDNA-5/FRT 3xFlag-MAEA	This study	N/A
pcDNA-5/FRT WDR26-HA (Isoform 2)	This study	N/A
pcDNA-3.1/Hygro HA-WDR26 (Isoform 2)	This study	N/A
pRSF GID4(Δ1-99)-6xHis	Sherpa et al.^25^	N/A
pRSF NMNAT1-6xHis	This study	N/A
pRSF GG-NMNAT1-6xHis	This study	N/A
pRSF NMNAT1 (Δ120-130)-6xHis	This study	N/A
pcDNA-5/FRT NMNAT1-HA	This study	N/A
pcDNA-5/FRT NMNAT1(Δ120-130)-HA	This study	N/A
pcDNA-5/FRT NMNAT1-GGGSGS-LERPGRKRKWT-GGGSGS-HA (Δ120-130) (Δbasic+C-basic)	This study	N/A
pcDNA-5/FRT NMNAT1-HA ((R125, K126, R127, K128, W129)>(P125, K126, K127, K128, R129, K130, V131)-HA (basic>NLS)	This study	N/A
pcDNA-3.1/Hygro YPEL5-HA	This study	N/A
pcDNA-3.1/Hygro YPEL5-HA (Δ2-5) (ΔN-term)	This study	N/A
pcDNA-3.1/Hygro NMNAT1-HA	This study	N/A
pcDNA-3.1/Hygro NMNAT1-HA (Δ120-130)	This study	N/A
pcDNA-5/FRT NMNAT1-GGGSGS-LERPGRKRKWT-GGGSGS-HA (Δ120-130) (Δbasic+C-basic)	This study	N/A
pcDNA-3.1/Hygro NMNAT1-HA ((R125, K126, R127, K128, W129)>(P125, K126, K127, K128, R129, K130, V131)-HA (basic>NLS)	This study	N/A
pRSF NMNAT1 (K126A/R127A/K128A/W129A)-6xHis	This study	N/A
pRSF NMNAT1 (Y64A/I68A/E71A/L88A/K250A/H251/A)-6xHis	This study	N/A
pRSF NMNAT1 (R125, K126, R127, K128, W129)>(P125, K126, K127, K128, R129, K130, V131)-6xHis (basic>NLS)	This study	N/A
pGEX GST-TEV-GID4(Δ1-99)	Sherpa et al.^25^	N/A
pGEX GST-3C-Ub	This study	N/A
pGEX GST-3C-Ub K0 (all K > R)	This study	N/A
pGEX GST-3C-Ub K48 (all K > R; R48K)	This study	N/A
pGEX GST-3C-Ub K48R (K48R)	This study	N/A
pGEX GST-TEV-UBE2H	Sherpa et al.^25^	N/A
pRSF CK2a-6xHis	Chrustowicz et al.^75^	N/A
pFLN WDR26	This study	N/A
pFLN YPEL5	This study	N/A
pET28a 6His-TEV-Halo-TUBE-4xUbiquilin1	MRC-PPU Reagents	Cat# DU23799
pET29 sortase A	Sherpa et al.^25^	N/A
pBABED-U6-PURO	gift from Thomas Macartney, University of Dundee, UK	N/A
px335-Cas9(D10N)	Addgene	Plasmid# RRID: Addgene_42335
pBIG2 RANBP9: TWA1-TEV 2xS: ARMC8: RMND5A: MAEA	Sherpa, et al.^25^	N/A
pBIG2 RANBP9: TWA1-TEV 2xS: RMND5A: MAEA	This study	N/A
		
**Software and Algorithms**		
Fiji/ImageJ	Schindelin et al.^76^	https://imagej.net/Welcome
GraphPad Prism version 9.4	GraphPad Software	https://www.graphpad.com
Focus	Biyani et al.^77^	https://lbem-focus.epfl.ch
SerialEM v3.8.0-b5	Mastronarde^78^	http://bio3d.colorado.edu/SerialEM/
EPU v2.7.0	Thermo Fisher Scientific	https://www.thermofisher.cn/cn/zh/home/electron-microscopy/products/software-em-3d-vis/epu-software.html
MotionCor2 v1.1	Zheng et al.^79^	https://emcore.ucsf.edu/ucsf-software
Gctf v1.06	Zhang et al.^80^	https://github.com/JackZhang-Lab/GCTF
Gautomatch v0.56	Kai Zhang (MRC LMB)	https://github.com/JackZhang-Lab/Gautmatch
Relion v4.0	Zivanov et al.^81^ and Scheres^82^	https://github.com/3dem/relion
UCSF Chimera v.1.13.1	Petterson et al.^83^	https://www.cgl.ucsf.edu/chimera/
UCSF ChimeraX v1.5	Petterson et al.^84^	https://www.cgl.ucsf.edu/chimerax/
PyMOL v2.5.2	Schrödinger	https://pymol.org/2/
Coot v0.9.8.7	Emsley and Cowtan^85^ and Emsley et al.^86^	https://www2.mrc-lmb.cam.ac.uk/personal/pemsley/coot/
PHENIX v1.19.2	Adams et al.^87^	https://www.phenix-online.org/
Molprobity	Chen et al.^88^	http://molprobity.biochem.duke.edu/
Image Studio	LI-COR Biosciences	https://www.licor.com/bio/image-studio/
ImageQuant	GE Healthcare	N/A
		
**Other**		
Lipofectamine LTX with Plus reagent	Invitrogen	Cat# 15338100
Lipofectamine 3000	Invitrogen	Cat# L3000015
Lipofectamine RNAiMAX	Invitrogen	Cat# 13778075
DMEM, high glucose	Gibco	Cat# 11965092
McCoy’s 5A Media	Gibco	Cat# 16600082
Glutmax	Gibco	Cat# 35050061
Penicillin-Streptomycin	Gibco	Cat# 15070063
Blasticidin S HCl	Gibco	Cat# A1113903
Puromycin	Gibco	Cat# A1113802
Zeocin	Gibco	Cat# R25001
Hygromycin B	Gibco	Cat# 10687010
Fetal Bovine Serum, Heat Inactivated	Gibco	Cat# 10438026
R1.2/1.3, Cu 200 mesh, holey carbon grids	Quantifoil	Cat#N1-C14nCu20-01
Monoclonal Anti-HA-Agarose (clone HA-7)	Sigma Aldrich	Cat# A2095-1ML

#### Lead contact

Further information and requests for resources and reagents should be directed to and will be fulfilled by the lead contact, Dr. Arno Alpi (aalpi@biochem.mpg.de)

#### Materials availability

All unique/stable reagents generated in this study are listed in the key resource table and are available from the lead contact with a completed Materials Transfer Agreement.

### Experimental Model And Study Participant Details

#### Cell lines

##### Flp_In-T-Rex-HEK293 Human Cells (RRID: CVCL_U427)

Flip-In T-Rex-HEK293 were obtained from Thermofisher Scientific and cultured in DMEM (GIBCO), supplemented with FBS (10% (v/v)) (GIBCO), GlutaMAX (GIBCO), penicillin (100 units/ml), streptomycin (0.1 mg/ml), Zeocin (100 µg/ml), and Blasticidin S HCl (15 µg/ml) (GIBCO) at 37°C in a humidified incubator at 7% CO_2_.

##### Flp_In-T-Rex-HeLa Human Cells (RRID: CVCL_C4ET)

Flip-In T-Rex-HeLa were a gift from Christian Behrends (Ludwig-Maximilians-University, Germany) and cultured in DMEM (GIBCO), supplemented with FBS (10% (v/v)) (GIBCO), GlutaMAX (GIBCO), penicillin (100 units/ml), streptomycin (0.1 mg/ml), Zeocin (100 µg/ml), and Blasticidin S HCl (15 µg/ml) (GIBCO) at 37°C in a humidified incubator at 7% CO_2_.

##### U2OS Human Cells (RRID: CVCL_0042)

U2OS cells were obtained from ATCC and cultured in DMEM (GIBCO), supplemented with FBS (10% (v/v)) (GIBCO), GlutaMAX (GIBCO), 0.1 mM Sodium Pyruvate (GIBCO), 100 units/ml penicillin, 0.1 mg/ml streptomycin, at 37°C in a humidified incubator at 7% CO_2_.

##### SH-SY5Y Human Cells (RRID: CVCL_0019)

SH-SY5Y cells were obtained from ATCC and cultured in DMEM (GIBCO), supplemented with FBS (10% (v/v)) (GIBCO), GlutaMAX (GIBCO), 0.1 mM Sodium Pyruvate (GIBCO), 100 units/ml penicillin, 0.1 mg/ml streptomycin, at 37°C in a humidified incubator at 7% CO_2_.

##### SW48 Human Cells (RRID: CVCL_1724)

SW48 (ATCC, CCL-231) cells were obtained from ATCC and cultured in DMEM (GIBCO), supplemented with FBS (10% (v/v)) (GIBCO), GlutaMAX (GIBCO), penicillin (100 units/ml), streptomycin (0.1 mg/ml) at 37°C in a humidified incubator at 7% CO_2_.

##### High Five Insect Cells

Cells were grown in EX-CELL 420 Serum-free Medium at 27°C with shaking at 130 rpm.

#### Organisms/Strains

##### E. coli BL21 RIL (DE3)

Cells were grown in Terrific Broth (TB) medium at 37 or 18°C with shaking at 180 rpm.

##### E. coli DH5α

Cells were grown in Luria-Bertani (LB) medium at 37°C with shaking at 180 rpm.

### Method Details

#### Cell biological experiments

##### Cell culture

Flip-In T-Rex-HEK293 (ATCC, CRL-1573) were obtained from Thermofisher Scientific and Flip-In T-Rex-HeLa were a gift from Christian Behrends (Ludwig-Maximilians-University, Germany) and cultured in DMEM (GIBCO), supplemented with FBS (10% (v/v)) (GIBCO), GlutaMAX (GIBCO), penicillin (100 units/ml), streptomycin (0.1 mg/ml), Zeocin (100 µg/ml), and Blasticidin S HCl (15 µg/ml) (GIBCO) at 37°C in a humidified incubator at 7% CO_2_. U2OS (HTB-96) and SH-SY5Y (CRL-2266) cells were obtained from ATCC and cultured in DMEM (GIBCO), supplemented with FBS (10% (v/v)) (GIBCO), GlutaMAX (GIBCO), Sodium Pyruvate (0.1 mM (GIBCO), penicillin (100 units/ml), streptomycin (0.1 mg/ml), at 37°C and 7% CO_2_. SW48 (ATCC, CCL-231) cells were obtained from ATCC and cultured in DMEM (GIBCO), supplemented with FBS (10% (v/v)) (GIBCO), GlutaMAX (GIBCO), penicillin (100 units/ml), streptomycin (0.1 mg/ml) at 37°C in a humidified incubator at 7% CO_2_. The cultures were frequently checked for the absence of mycoplasma contamination.

##### CRISPR-Cas9 edited HEK293 and HeLa cells

*MAEA*^*-/-*^, *WDR26*^*-/-*^, and *YPEL5*^*-/-*^ deficient cells were generated using CRISPR-Cas9(D10A) nickase genome editing strategy. ATUM gRNA design tool was utilized to identify paired sense and antisense guide RNAs (gRNA) targeting exon 2 of *MAEA*, exon 1 of *WDR26*, and exon 2 of *YPEL5*^25^ (Figure S1A-C). Sense and antisense gRNA were cloned into pBABED-U6-Puromycin plasmid (gift from Thomas Macartney, University of Dundee, UK) and pX335-Cas9(D10A) (Addgene), respectively. Flp-In T-Rex-HEK293 and Flp-In T-Rex-HeLa Cells cells were co-transfected with sense/antisense gRNA plasmids using Lipofectamine 3000 (Invitrogen) transfection reagent following manufacturers protocol. 24 hours after transfection, cells were selected in puromycin (2 μg/ml) for two days, followed by expansion, and single-cell dilution to obtain cell clones. Successful knockout clones were verified by immunoblotting and genomic sequencing of targeted loci (Figure S1D-F).

To generate rescue cell lines *resMAEA*^*-/-*^ and *resWDR26*^*-/-*^ N-terminally 3xFlag-tagged MAEA and N-terminally HA-tagged WDR26 was reintroduced respectively, using the Flp-In system (Invitrogen). Expression of stably integrated 3xFlag-MAEA and HA-WDR26 was induced with 1 µg/ml of tetracycline overnight prior to any performed experiment.

##### CRISPR-Cas9 edited SW48 cells

gRNA for human *YPEL5* and *WDR26* were ligated into lentiCRISPR v2 (Addgene #52961) using BsmBI restriction sites. Together with the lentiviral envelope plasmid psPax2 (Addgene #12260) and the packaging plasmid pMD2.G (Addgene #12259), two gRNA plasmids containing sense and antisense gRNA were transfected with Lipofectamine 3000 (Invitrogen) into HEK cells to produce viral particles. After 48 hours incubation, the supernatant containing the viral particles was collected. SW48 cells were seeded in 6-well format, complemented with 8 µg/mL polybrene, and virus-containing supernatant added to the cells. After 24 hours selection for targeted cells were initiated with 3 µg/mL puromycin and incubated for further 72 hours. Pool of puromycin-resistant WDR26sg- and YPEL5sg-targeted SW48 cells were confirmed by immunoblotting.

##### siRNA-mediated knockdown of CTLH subunit

siRNA transfection of Flp-In T-Rex-HEK293, SW48, Flp-In T-Rex-HeLa, SH-SY5Y, and U2OS cells were carried out using Lipofectamine RNAiMAX (Thermofisher) reagents following manufacturers protocol for forward transfection. Used siRNA: ON-TARGETplus Non-targetting control, ON-TARGETplus SMARTpool Human-YPEL5 (GE Dharmacon), Mission esiRNAs targeting human *MAEA, WDR26, YPEL5*, and *NMNAT1* (Sigma). Briefly, Lipofectamine RNAiMAX was diluted 50 times in OptiMEM, mixed with equal volume of 0.1 µM siRNA in OptiMEM, incubated at room temperature for 10 min, and added dropwise to the cells (10 nM final siRNA concentration). Cells were incubated for 48-96 hours at 37 °C before performing further analysis.

##### Transient expression in mammalian cells

cDNA of CTLH subunits, NMNAT1, and HBP1 were cloned into pcDNA-5 FRT/TO-Hygro or pcDNA3.1-Hygro expression vectors using standard molecular biology techniques and constructs were verified by DNA sequencing. For transient expressions, HEK293 cells were transfected with 5 µg plasmid, using 0.5 mg/ml of Polyethylenimine (PEI) solution (stock of 1 mg/ml dissolved in 0.2 M HCl) in serum free media, followed by supplementing serum after 3 hours of transfection and cultured for 36 hours before further analysis.

##### TUBE-enrichment

We adapted a Tandem Ubiquitin Binding Entities (TUBEs) approach to enrich ubiquitylated proteins. The pan-ubiquitin binding TUBE, 6xHis-Halo-4xUBQLN1 (Halo-TUBE), was expressed in *E coli* BL21 cells, affinity purified with His-select Nickel Affinity Gel (Sigma), and dialyzed into 50 mM Tris-HCL pH 7.5, 150 mM NaCl, and 1 mM DTT, followed by size exclusion chromatography (SEC).

21 µmol recombinant Halo-TUBE was equilibrated with 200 µl HaloLink resin (Promega) in 1 ml Halo binding buffer (HBB, 50 mM Tris/HCl pH 7.5, 150 mM NaCl, 0.05% NP-40, 1 mM DTT) over night at 4°C, Halo-TUBE-coupled resin was washed with HBB and used within a week for pull down experiments. To enrich for ubiquitylated proteins, cells were harvested, washed twice with Phospate-Buffered saline (PBS), resuspended in lysis buffer (LB, 50 mM Tris/HCl pH 7.5, 150 mM NaCl, 1% NP-40, 1 mM EGTA, 1 mM EDTA, 0.27 M Sucrose, 5mM NEM (N-ethylmaleimide), and HALT protease/phosphatase inhibitor cocktail [Thermo Fisher Scientific]), and homogenized by pushing 15x through a 22G syringe. Lysates were cleared by centrifugation at 4°C, 20000 x g for 30 min. For MS-based proteome analysis, 1mg of cell lysate were incubated with 15 µl of Halo-TUBE resins for two hours at 4°C. Halo-TUBE precipitates were extensively washed with LB supplemented with 0.5 M NaCl followed by two additionally washed with detergent free LB. For immunoblot analysis, 3 mg of cell lysates, supplemented with 5 mM N-ethylmaleimide (NEM) or 1 µM OTUB1 where indicated, were incubated with 20 µl of Halo-TUBE resins for two hours at 4°C. Halo-TUBE precipitates were washed with LB supplemented with 0.5 M NaCl and eluted by boiling in reducing SDS sample buffer, separated on SDS-PAGE followed by immunoblot analysis.

For detecting ubiquitinated species of NMNAT1, C-terminally HA tagged NMNAT1 (wildtype and mutants as indicated) were transiently transfected in Flip-In T-Rex-HEK293 cells for 36 hours and proceeded for TUBE enrichment as described above.

##### Immunoprecipitation

C-terminally HA-tagged YPEL5 or NMNAT1 (wildtype and mutants as indicated) were transiently transfected in Flip-In T-Rex-HEK293 cells for 36 hours. Cells were harvested and washed twice with PBS, resuspended in IP lysis buffer (IP-LB: 40 mM HEPES pH 7.5, 120 mM NaCl, 1% TX-100, HALT Protease Inhibitor (PIERCE), 1 mM DTT, and Benzonase 10U/ml), and homogenized by pushing 15x through a 22G syringe. Lysates were cleared by centrifugation at 20,000 × g for 30 min at 4°C. 3 mg of lysates were incubated with Lysis-IP buffer-equilibrated Anti-HA-Agarose beads (Sigma Aldrich) for 2 hours at 4°C. HA-immunoprecipitates were extensively washed with Lysis-IP buffer and eluted by boiling in reducing SDS sample buffer, separated on SDS-PAGE followed by immunoblot analysis.

For detection of NMNAT1 *in vivo* ubiquitylation (Figure 4C), NMNAT1-HA transfected Flip-In T-Rex-HEK293 cells were treated with 1 µM MG132 for 12 hours and HA-IP was carried out as described above. HA-immunoprecipitates were resuspended in 150 ul resuspension buffer (RB: 50 mM Tris/HCl pH 7.5, 150 mM NaCl, 0.05% NP-40, 1mM DTT), split in two halfs and either mock-treated or incubated with 1 µM OTUB1 at 30°C for two hours. Reactions were terminated with SDS sample buffer, separated on SDS-PAGE followed by immunoblot analysis.

##### Cell lysates, treatments, and immunoblot analysis

Flp-In T-Rex-HEK293 cell lines were washed once with PBS, lysed in RIPA buffer (Cell Signaling), supplemented with cOmplete Protease Inhibitor Mix (Roche), HALT protease/phoshotase inhibitor cocktail (Thermo Fisher Scientific), Benzonase 10 U/ml (Milipore) and homogenized by pushing 10x through a 22G syringe. Lysates were cleared by centrifugation at 20,000 × g for 20 min at 4°C, and protein concentration determined by Micro BCA-Protein Assay (Thermo Fisher Scientific).

For immunoblot analysis, equal amounts of lysates were separated on SDS-PAGE, and proteins were visualized by immunoblotting using indicated primary antibodies: NMNAT1 (Santa Cruz, sc-271557), MAEA (R&D Systems, AF7288), RANBP9 (Abnova, PAB16671), ARMC8 (Santa Cruz, sc-365307), WDR26 (abcam, ab85962), MKLN1 (Santa Cruz, sc-398956), YPEL5 (Thermo Fisher Scientific, PA5-26957), Ubiquitin clone FK2 (Sigma, 04-263), β-Actin (Cell Signaling, #4970), Vinculin (abcam, ab129002), LC3B (Cell signaling, #2775). Immunoblots were developed using Clarity Western ECL Substrate (Bio-Rad) and imaged using Amersham Imager 600/800 (GE Lifesciences). For quantitation of immunoblots, at least three biological repetitions were performed, bands of scanned blots quantified using ImageJ software, and quantified signals normalized to loading control (Vinculin or Actin) as indicated. For statistical analysis, one-way ANOVA with Dunnett’s multiple comparison tests were performed and presented using Graphpad Prism software.

##### Subcellular fractionation of HEK293 and SW48

Cells were harvested, washed once with PBS and lysed in hypotonic buffer (HYPB: 10 mM Tris-HCl pH 7.4, 10 mM KCl, 1.5 mM MgCl_2_, 10 mM 2-mercaptoethanol) by pushing through a 23G needle. Nuclei were collected by centrigugatio (2,700g, 10 s). Supernatant (consisting of the cytosolic fraction) were further cleared by centrifugation (16,000g, 20 min). Nuclei were washed twice with HYPB and nuclear proteins were extracted with high-salt buffer (15 mM Tris-HCl, pH 7.4, 1 mM EDTA, 500 mN NaCl, 1mM MgCl_2,_ 10% glycerol, 10 mM 2-mercaptoethanol, and cOmplete Protease Inhibitor Mix (Roche)). Fractions with comparable amounts (relative to each fraction) were separated by SDS-PAGE and analyzed by immunoblot analysis.

##### Tiazofurin treatment and cell viability assay

Flp-In T-Rex-HEK293 parental and knockout cell lines, or siRNA transfected Flp-In T-Rex-HEK293 and SW48 cells, were treated with tiazofurin (stock 100 mM in DMSO) (BLD Pharma, BD154096) in a concentration range from 475 to 14.5 µM by 2-fold serial dilutions as indicated. Cells were allowed to grow under drug treatment for 96 hours and their viability were measured using Cell Titer-Glo luminescent assay (Promega, G7570) following manufacturer’s instructions. Briefly, 1 volume of premixed Glo reagent was added to 4 volumes of cell culture and incubated at 37°C for 30 min. Luminescence was measured at 560 nm in a Clariostar plus plate reader (BMG Labtech company).

##### Proteosome inhibitor and cycloheximide treatments

Flip-In T-Rex-HEK293 cell lines were treated with 10 µM MG132 proteosome inhibitor (Sigma, M8699, stock: 42 mM in DMSO) or mock for 12 hours followed by cell lysis and immunoblot analysis. To block translation, indicated cell types were treated with 300 µg/ml of cycloheximide (Sigma, #01810, stock 100 mg/ml in DMSO) for 0-9 hours and subjected to cell lysis and immunoblot analysis.

#### Plasmid preparation and mutagenesis

The genes coding for human CTLH complex subunits were obtained from human cDNA library (Max Planck Institute of Biochemistry) except for GID4, which was codon-optimized for bacterial expression and synthesized by GeneArt gene synthesis service (Thermo Fisher Scientific).^25^ The NMNAT1 cDNA for recombinant protein expression was synthesized by TWIST Bioscience. Plasmids for protein expression were prepared using Gibson assembly method^89^ and mutant versions were either generated by QuickChangeII protocol (Stratagene) or synthesized by TWIST bioscience. To generate NMNAT1^bp>NLS^, the basic motif amino acid sequence R125, K126, R127, K128, W129 was replaced with the canonical nuclear localization signal (NLS) of SV40 virus: P125, K126, K127, K128, R129, K130, V132. For insect cell expression of CTLH E3 complex assemblies, genes of CTLH subunits were combined into single baculoviral expression constructs using the biGBac method.^90^ All constructs were verified by DNA sequencing and mutants were checked by intact-mass spectrometry.

#### Protein preparation

##### Insect cell expression and purification

The human CTLH complexes: core-CTLH (comprising RANBP9-TWA1(2xS)-RMND5A-MAEA-ARMC8α), WDR26-CLTH (core-CTLH with WDR26), WDR26-YPEL5-CTLH (core complex with WDR26 and YPEL5), and MKLN1-CLTH (core-CTLH with MKLN1) were expressed and purified from High Five insect cells as described previously.^25^ Briefly, cell pellets were resuspended in 50 mM Tris/HCl pH 7.5, 150 mM NaCl, 5 mM DTT, 10 mg/ml leupeptin, 20 mg/ml aprotinin, EDTA-free cOmplete protease inhibitor tablet (Roche, 1 tablet/50 mL of buffer), and 1 mM PMSF. CTLH complexes were purified from lysates by Strep-Tactin affinity chromatography of C-terminally 2xStrep tagged TWA1 followed by a SEC using Superose 6 column in the running buffer of 25 mM HEPES pH 7.5, 150 mM NaCl, and 5 mM (for cryo-EM, buffer A) or 1 mM DTT (for biochemical assays, buffer B). GST-tagged MKLN1, WDR26, YPEL5, and GID4 were purified by Glutathione Sepharose 4B affinity chromatography, followed by anion exchange (0-1 M NaCl, 25mM Tris pH 7.5, 5mM DTT) and SEC in buffer B.

GST-tagged UBE2H was expressed in High Five insect cells and pellet was resuspended in 50 mM Tris/HCl pH 7.5, 150 mM NaCl, 5 mM DTT, 10 mg/ml leupeptin, 20 mg/ml aprotinin, EDTA-free Complete protease inhibitor tablet (Roche, 1 tablet/50 mL of buffer), and 1 mM PMSF. UBE2H protein was affinity captured by Glutathione Sepharose 4B resin followed by overnight digestion at 4°C with tobacco etch virus (TEV) protease to remove the GST tag. The protein was then further purified using anion exchange chromatography (0-1 M NaCl, 25 mM Tris/HCl pH 7.5, and 5 mM DTT), SEC in buffer B, and passed-back over Glutathione Sepharose 4B resin to remove uncleaved GST-UBE2H and free GST.

##### Bacterial expression and purification

C-terminal 6xHis-tagged NMNAT1 wildtype and mutant versions, as listed in the key resources table, were expressed in *E. coli* (DE3) RIL cells, and cell pellets were resuspended in 50 mM MES pH 6.5, 20 mM imidazole, 150 mM NaCl, 1 mM PMSF, and 5 mM beta-mercaptoethanol. Proteins were purified by His-select Nickel Affinity Gel chromatography (Sigma Aldrich), and eluted in 300 mM imidazole, followed by a cation exchange chromatography (0-1 M NaCl, 25mM MES pH 6.5, 5mM DTT) and SEC in buffer B.

C-terminal His_6_-tagged GID4(Δ1-99) was expressed in *E. coli* (DE3) RIL cells, cell pellets were resuspended in 50 mM Tris/HCl pH 7.5, 20 mM imidazole, 150 mM NaCl, 1 mM PMSF, and 5 mM beta-mercaptoethanol. Proteins were purified by His-select Nickel Affinity Gel chromatography (Sigma), and eluted in 300 mM imidazole, followed by SEC in buffer B. Mutant versions of ubiquitin were expressed in *E. coli* (DE3) RIL cells, cell pellets were resuspended in 50 mM Tris/HCl pH 7.5, 150 mM NaCl, 5 mM DTT, and 1 mM PMSF. The ubiquitin mutants were captured by affinity chromatography using Glutathione Sepharose 4B resin followed by overnight on-beads digestion at 4°C with human rhinovirus 3C (HRV 3C) protease to remove the GST-tag. Eluted proteins were subjected to anion exchange chromatography (0-1 M NaCl, 25 mM Tris/HCl pH 7.5, and 5 mM DTT), followed by SEC in buffer B.

For the fully phosphorylated UBE2H used for the determination of the preferred ubiquitin lysine, a GST-tagged version of UBE2H was co-expressed with the catalytic subunit of CK2 kinase (CK2α) in BL21 (DE) PRIL *E. coli* and purified as described recently.^75^ Briefly, cell pellets were resuspended in 50 mM Tris/HCl pH 7.5, 150 mM NaCl, 5 mM DTT, and 1 mM PMSF, and UBE2H purified by glutathione affinity chromatography (Cytiva), followed by overnight on-beads digestion at 4°C with human rhinovirus 3C (HRV 3C) protease to elute UBE2H from the GST-tag. Eluate was further subjected to anion exchange chromatography and SEC in buffer B. To remove residual GST, pooled fractions were passed back over the glutathione affinity resin. Phosphorylation of UBE2H was verified by intact mass analysis performed in the MPIB Mass Spectrometry Core Facility.

Untagged wildtype and lysine mutant variants of ubiquitin were expressed in BL21 (DE) PRIL *E. coli* and purified via glacial acetic acid method as described previously.^91^ Cells were resuspended in a lysis buffer consisting of 50 mM Tris/HCl pH 7.5, 150 mM NaCl, 10% (v/v) glycerol, 1% (v/v) Triton X-100, 1 mM EDTA and cOmplete Protease Inhibitor Mix (Roche). Resuspended cells were lysed by sonication, and lysate was cleared by centrifugation at 40,905*g* for 60 min. Glacial acetic acid (Sigma Aldrich) was added dropwise to the lysate with mixing until the solution reached pH 4. Ubiquitin-containing supernatant was cleared by centrifugation at 20,199*g* for 20 min and dialyzed into 25 mM sodium acetate (pH 4.5) over night. Ubiquitin was further purified by gravity S column cation exchange chromatography and SEC in buffer B.

##### Fluorescent labeling

Fluorescent version of GG-NMNAT1-His_6_ was generated using sortase A-catalyzed reaction by fusing its N-terminus with ^TAMRA^LPETGG peptide.^92^ The reaction mixture containing 150 µM TAMRA peptide, 10 µM NMNAT1, 20 µM sortase A in the reaction buffer (50 mM Tris/HCl pH 8.0, 150 mM NaCl and 10 mM CaCl_2_) was incubated at RT for 30 min. Finally, SEC in buffer B was performed to remove sortase A and unreacted peptide.

#### *In vitro* protein binding assays

To test NMNAT1 binding to individual CTLH subunits, 2.5 µM of recombinant GST-fusions of GID4, MKLN1, WDR26, and were incubated with 10 µM NMNAT1 in buffer B (50 mM HEPES pH 7.5, 150 mM NaCl, and 1 mM DTT) at 4°C for two hours. Protein complexes were captured with Glutathione Sepharose 4B resin (Cytiva) by incubation at 4°C for further two hours. Resin was washed five times with buffer B followed by digestion at 16°C with 0.3 µM Tobacco Etch Virus (TEV) protease overnight. The TEV-eluted samples were boiled in reducing SDS sample buffer, separated on SDS-PAGE followed by Coomassie staining or immunoblot analysis.

The NMNAT1(wildtype and mutant versions) binding with different CTLH complexes (core- and WDR26-CTLH) were tested by incubating 200 μg of NMNAT1 (hexamer) with equal amounts of CTLH complexes in 300 µl buffer B for 30 min on ice. Protein complexes were captured by adding Strep-Tactin resin, incubating at 4°C for 45 mins, and thoroughly washing three times with buffer B. The pull-down fractions were then separated on SDS-PAGE and visualized by Coomassie staining.

#### *In vitro* ubiquitylation assays

Ubiquitylation reactions were performed in reaction buffer (RB, 25 mM HEPES pH 7.5, 150 mM NaCl, 5 mM ATP, and 10 mM MgCl_2_) by mixing 0.2 µM UBA1, 2 µM UBE2H, 0.5 µM CTLH E3 ligase complex, 1 µM GID4(Δ1–99), 2 µM fluorescent peptide (C-terminally fluorescently labelled peptide containing N-terminal PGLW GID4 interacting sequence^25^) or 40 nM (calculated as hexamer) fluorescent TAMRA-tagged NMNAT1 together with 20 µM ubiquitin. Reactions were quenched with SDS loading buffer and visualized by a fluorescent scan of SDS-PAGE gel using the Amersham Typhoon imager (GE Healthcare). *In vitro* ubiquitylation assays assessing NMNAT1 mutants were carried out in a similar manner, but using 200 nM of non-labelled wildtype and mutant NMNAT1 as substrates, and NMNAT1 ubiquitin conjugates visualized by immunoblot analysis using NMNAT1 antibody (SantaCruz, sc-271557). *In vitro* ubiquitylation assays with ubiquitin variants were performed in RB as describe above, except 1 μM of CTLH E3 complex without ARMC8 and GID4 was used.

#### Cryo-EM experiments

##### Cryo-EM sample preparation and imaging

To prepare cryo-EM samples of the WDR26-CTLH E3 (comprising RANBP9-TWA1-RMND5A-MAEA-WDR26) bound to NMNAT1, the affinity-purified complex was incubated with 4-fold molar excess of the substrate (NMNAT1-6xHis) for 30 min on ice. The mixture was subjected to SEC (Superose 6 column) in buffer A to separate an unbound pool of NMNAT1.

For cryo-EM of WDR26-CTLH E3 (comprising RANBP9-TWA1-RMND5A-MAEA-WDR26) bound to YPEL5, all proteins were co-expressed in insect cells and purified by StrepTactin-affinity chromatography, pulling on a Twin-Strep tag fused to TWA1 C-terminus. Pull-down fractions were run on SEC (Superose 6 column) in buffer A.

For both cryo-EM samples, the SEC-purified complexes were concentrated to 1.6-3 mg/ml and supplemented with 0.01% octyl-β-Glucoside right before plunging.

Cryo-EM grids were prepared using Vitrobot Mark IV (Thermo Fisher Scientific) operated at 4°C and 100% humidity. 3 µl of samples were applied to glow-discharged Quantifoil holey carbon grids (R1.2/1.3 200 mesh). Grids were blotted with Whatman no. 1 filter paper (blot time: 3 s, blot force: 3) and vitrified by plunging into liquid ethane.

Initial low-resolution cryo-EM datasets were collected on a Glacios transmission electron microscope (Thermo Fisher Scientific) operated at 200 kV, equipped with a K2 direct electron detector (Gatan). The screened grids were used to collect high-resolution datasets on a Titan Krios microscope (Thermo Fisher Scientific) operated at 300 kV, equipped with a post-column GIF and a K3 Summit direct electron detector (Gatan) operating in a counting mode. SerialEM^93^ was used for screening the grids and automated data collection. Details of data collection and map refinement are summarized in Table S3 and Figures S4 and S6.

##### Cryo-EM data processing

Movie frames were motion-corrected with dose weighting using MotionCor2^79^ and subjected to estimation of contrast transfer function parameters with Gctf v1.06^80^ integrated in Relion v4.0^81,82^ or Focus software^77^ (used for on-the-fly pre-processing of Titan Krios data, while also automatically discarding poor quality images). Particles were automatically picked with Gautomatch (K. Zhang, MRC Laboratory of Molecular Biology, Cambridge, UK) using a previously published map of the supramolecular WDR26-CTLH assembly (EMD-12542) as a template. All subsequent stages of data processing were carried out with Relion. To clean up the data while preserving rare views, extracted particles were subjected directly to unmasked 3D classification.

###### • NMNAT1-bound WDR26-CTLH E3 (Figure S4)

A clean set of particles selected after two rounds of unmasked 3D classification was further 3D classified, revealing co-existence of two types of NMNAT1-bound WDR26-CTLH E3 assemblies with similar shapes and dimensions but distinctive stoichiometry and configuration of the constituent modules: assembly I containing two opposing catalytic modules connected by two WDR26-RANBP9-TWA1 units (Figure S3A); assembly II – not observed previously, wherein one catalytic module is replaced by an extra RANBP9-TWA1-WDR26 unit (Figure S3B). Additional rounds of 3D classifications and 3D refinements segregated particles into an assortment of NMNAT1-bound complexes: 2 classes of assembly I with the same mode of NMNAT1 binding but distinct orientations of catalytic modules; 3 classes of assembly II with NMNAT1 core binding to either one of the three (class 2 and 3) or simultaneously to two WDR26 dimers (class 1).

To determine molecular details of NMNAT1 capture, particles after initial 3D classifications were directly subjected to 3D refinement, yielding a map of WDR26-CTLH E3 assembly containing a weak electron density of centrally bound substrate. To enrich for particles with encapsulated NMNAT1, a focused refinement over the NMNAT1^core^-bound RANBP9-TWA1-WDR26 module was performed, followed by focused 3D classification with a mask around NMNAT1. Particles from the best-resolved class were subjected to 3D refinement with a two-fold symmetry (C2) imposed, resulting in a map of WDR26-CTLH assembly I containing strong NMNAT1 density contacting propellers from two opposing WDR26 dimers. Masking out the catalytic modules and performing focused refinement over one NMNAT1-bound part of the complex showed a weak electron density corresponding to NMNAT1^basic^ extending towards WDR26 β-propellers. A series of focused 3D classifications and local refinements with masks around the opposite side of the complex yielded a high-resolution reconstruction of NMNAT1 core-bound WDR26.

###### • YPEL5-bound WDR26-CTLH E3 (Figure S7)

Particles after two rounds of 3D classification were first subjected to another 3D classification (with a higher value of the T parameter), which revealed classes corresponding to WDR26-CTLH assembly I and II (as described above), in which all copies of WDR26 modules were engaged by YPEL5 molecules (Figure S7A).

To obtain a high-resolution map of YPEL5-bound WDR26, the clean set of particles was directly refined with a C2 symmetry imposed. The overall map of the complex was used to generate masks over two opposing YPEL5-WDR26-RANBP9-TWA1 units. To duplicate the particle number during subsequent processing steps, the generated masks were used for signal subtraction, thus yielding two particle pools, which were combined and aligned by 3D refinement (as described previously for determining the structure of yeast Chelator-GID^SR4^).^25^ A series of local 3D classifications and focused refinements with progressively smaller masks over the YPEL5-bound WDR26 resulted in the final high-resolution reconstruction.

All maps were post-processed by B-factor sharpening and high-resolution noise substitution in Relion. In addition, to aid in building atomic models, the refined high-resolution maps were sharpened with DeepEMhancer^94^ and are deposited as additional maps in EMDB. The estimated resolutions of all reconstructions are based on the gold-standard Fourier Shell Correlation (FSC) at 0.143 cut-off. Simplified flow charts of cryo-EM data processing are presented in Figures S4 and S6.

##### Model building and refinement

Manual building of structural models was performed with Coot.^85,86^ The analysis and visualization of structures were carried out with UCSF Chimera v1.13.1,^83^ UCSF ChimeraX v1.5^84^ and PyMOL v2.5.2 (Schrödinger). Parameters of the built models are listed in Table S3.

###### • NMNAT1 core-bound WDR26 dimer

The crystal structure of NMN-bound NMNAT1 (PDB 1GZU) and AlphaFold2 predictions^95^ of WDR26 (split into individual domains) were fit into the post-processed map and manually refined. The characteristic ‘cross-brace’ arrangement of WDR26’s cysteine and histidine sidechains within its homodimerization domain supported fitting a previously unannotated zinc ion into a central connecting electron density, which is not present in the orthologous yeast GID subunit Gid7 (PDB 7NSB). The coordinated zinc glues together parts of the loop between CRA^C^ and β-propeller domains of WDR26, which appears to stabilize their relative orientations (Figure S5E). Moreover, we observed strong densities in the active sites of NMNAT1 protomers interacting with WDR26 propellers through the catalytic core domains. These densities accommodate coordinates for NMNAT1 substrate nicotinamide mononucleotide (NMN) from the previous NMNAT1 crystal structure (PDB 1GZU) used as an initial model for structure building (Figure S5D). Although NMN fits well in the extra densities, we cannot exclude the possibility that the bound molecule might correspond to the NMNAT1 product NAD^+^, whose NMN moiety is superimposable on the NMNAT1-bound NMN (Figure S5F). In such a scenario, the adenylyl phosphate portion of NAD^+^ would not be visible due to its positional flexibility that has been previously described.^96^ Since neither NMN nor NAD^+^ were present in the buffers used for protein purification, we presume one or the other, or a mixture of both, co-purified with NMNAT1 from E. coli, as was previously reported in the structural study of a bacterial NMNAT1^97^.

###### • YPEL5-bound WDR26 dimer

The cryo-EM model of WDR26 (from its NMNAT1-bound structure determined in this study) and AlphaFold2-prediction of YPEL5 were fit into a post-processed map and manually refined. The higher-resolution map of the YPEL5-bound complex confirmed the position of zinc ions in the structure of the WDR26 dimer. Moreover, coordinates for another, previously annotated zinc (UniProt ID: P62699) stabilizing YPEL5 fold were built in a prominent electron density located between sidechains of four centrally facing cysteine residues. A segment of unidentified peptide-like electron density in the hydrophobic cavity of the central YPEL5 β-sheet was left unassigned.

Structural models were subjected to iterative rounds of manual building and real space refinement in PHENIX^87^ until a satisfactory quality, in terms of geometry and agreement with the cryo-EM maps, was achieved. Configurations of the zinc-binding sites within WDR26 and YPEL5 were restrained during real-space refinement.

#### Mass spectrometry proteomics

##### Sample preparation

TUBE based enriched proteins were alkylated, reduced, and digested simultaneously using an SDC Buffer (1% Sodium deoxycholate (wt/vol) in 100 mM Tris pH 8.5) with 10 mM Tris(2-carboxyethyl)phosphine (TCEP), 40 mM 2-Chloracetamide (CAA) and 1 µg of Trypsin (Sigma-Aldrich) at 37°C overnight with agitation (1500 rpm) on an Eppendorf Thermomixer C. Peptides were desalted using SDB-RPS (Empore) StageTips. In brief, samples were fourfold diluted using 1% TFA in isopropanol and then loaded onto the StageTips, which were subsequently washed once with 1% TFA in isopropanol and then twice with 0.2% TFA/2% acetonitrile (ACN) twice. Peptides were eluted with 80% ACN/1.25% NH_4_OH and dried using a SpeedVac centrifuge (Concentrator Plus; Eppendorf) at 30 °C. Peptides were resuspended with in 0.2% TFA/2%. 200 ng of peptides were subjected to LC-MS/MS analysis.

##### Data-dependent acquisition (DDA) LC-MS analysis

Peptides were loaded on a 50 cm reversed phase column (75 μm inner diameter, packed in house with ReproSil-Pur C18-AQ 1.9 μm resin). To maintain a column temperature of 60 °C, we used a homemade column oven. An EASY-nLC 1200 system (Thermo Fisher Scientific) was connected online with a mass spectrometer (Orbitrap Exploris 480, Thermo Fisher Scientific) via nano-electrospray source. Peptides were separated using a binary buffer system consisting of buffer A (0.1% formic acid (FA)) and buffer B (80% ACN, 0.1% FA). We used a constant flow rate of 300 nl/min. We loaded 200 ng of peptides and eluted them with a 60 min gradient. For DDA LC-MS analysis we used a gradient that starts with 5% buffer B and increases consistently to 30% in 35 min, until it reaches 65% in 40 min and eventually 95% in 50 min. In the remaining 10 min buffer B decreases to 5%. DDA data was acquired with a full scan range of 300–1650 m/z at 60,000 resolution, automatic gain control (AGC) of 3e6 and a maximum injection time of 25 ms. The higher-energy collision dissociation (HCD) was set to 28. Each full scan was followed by 12 DDA scans which were performed at a 15,000 resolution, an AGC of 1e5 and the maximum injection time set to 28 ms. For DIA LC-MS analysis we used a gradient that starts with 5% buffer B and increases consistently to 30% in 45 min, until it reaches 95% in 55 min and remains constant for another 5 min. DIA data was acquired with a full scan range of 300–1650 m/z at 120,000 resolution, automatic gain control (AGC) of 3e6 and a maximum injection time of 60 ms. The higher-energy collision dissociation (HCD) was set to 28. Each full scan was followed by 32 DIA scans which were performed at a 30,000 resolution, an AGC of 1e6 and the maximum injection time set to 54 ms.

##### Data-independent acquisition (DIA) LC-MS analysis

Peptides were loaded on a 50 cm reversed phase column (75 μm inner diameter, packed in house with ReproSil-Pur C18-AQ 1.9 μm resin). To maintain a column temperature of 60 °C, we used a homemade column oven. An EASY-nLC 1200 system (Thermo Fisher Scientific) was connected online with a mass spectrometer (Orbitrap Exploris 480, Thermo Fisher Scientific) via nano-electrospray source. Peptides were separated using a binary buffer system consisting of buffer A (0.1% formic acid (FA)) and buffer B (80% ACN, 0.1% FA). We used a constant flow rate of 300 nl/min. We loaded 200 ng of peptides and eluted them with a 60 min gradient. The gradient starts with 5% buffer B and increases consistently to 30% in 45 min, until it reaches 95% in 55 min and remains constant for another 5 min. The MS data was acquired using a data independent acquisition (DIA) mode with a full scan range of 300–1650 m/z at 120,000 resolution, automatic gain control (AGC) of 3e6 and a maximum injection time of 60 ms. The higher-energy collision dissociation (HCD) was set to 28. Each full scan was followed by 32 DIA scans which were performed at a 30,000 resolution, an AGC of 1e6 and the maximum injection time set to 54 ms.

##### Data processing and bioinformatics analysis

DDA raw files were analyzed using^98^ version 1.6.7.0. Both searches were done against UniProt human reference proteome of canonical and isoform sequences with 42,347 entries for final protein identification and quantification. Enzyme specificity was set to trypsin with up to two missed cleavage-sites. Maximum and minimum peptide length was set to 25 to 7 respectively. Maximum number of variable modifications was set to two. The search included carbamidomethylation as a fixed modification and oxidation of methionine and GlyGly sites of Lysine as variable modifications. The rest of the settings were set to default.

DIA raw files were analyzed using library free search in DIA-NN version 1.8.1^99^. The search was done against UniProt human reference proteome of canonical and isoform sequences with 42,347 entries for final protein identification and quantification. Enzyme specificity was set to trypsin with up to one missed cleavage site. Maximum and minimum peptide length was set to 30 and 7. Maximum number of variable modifications was set to one. The search included carbamidomethylation as a fixed modification and oxidation of methionine and N-terminal acetylation of proteins as variable modifications. FASTA digest for library-free search/library generation, Deep learning-learning based spectra, RTs and IMs prediction and heuristic protein inferences was turned on. The precursor FDR were set to 1%. The rest of the settings were set to default.

The bioinformatics analyses and visualization were done using Python version 3.5.5 with the following packages: pandas 1.4.2, numpy 1.21.5, matplotlib 3.5.13, seaborn 0.11.2, scipy 1.7.3, statsmodels 0.13.2, scikit-learn 1.0.2, adjusttext 0.7.3. First, protein intensities were log2-transformed. Next, the dataset was filtered by a minimum of three valid values in at least one experimental group and subsequently imputed using a Gaussian normal distribution (width = 0.3 and downshift = 1.8). Student’s *t*-test was performed for determining the statistical significance. The Benjamini Hoechberg method was used for p-value correction (FDR). Hierarchical clustering was performed using the Euclidian distance. Data derived from MG132 proteasome inhibitor treatment and subsequent MS based proteomics analysis revealed no additional CTLH substrate candidate and were excluded from further analysis (Figure S2). After exclusion, the same downstream analysis mentioned above (filtering data for valid values, imputation, and Student’s *t*-test) was performed.

### Quantification And Statistical Analysis

For the quantitative assessment of cellular protein amounts, NMNAT1, MAEA, WDR26, YPEL5, and the loading controls (Actin and Vinculin) were visualized by immunoblot analysis and imaged using Amersham Imager 600/800 (GE Lifesciences). Signals of immunoblots were quantified using ImageJ software, and normalized to loading controls. For statistical analysis at least two biological replicates were considered, and one-way ANOVA with Dunnett’s multiple comparison tests were performed and plotted in PRISM v9.1.0 (GraphPad).

Cell viability was assessed and quantified using Cell Titer-Glo luminescent assay (Promega, G7570). Luminescence was measured at 560 nm in a Clariostar plus plate reader (BMG Labtech company) and relative viability calculated and plotted in PRISM v9.1.0 (GraphPad). For statistical analysis, at least three biological replicates were considered.

## Supplementary Material

Supplementary figures

Supplementary Tables

Data table S3

## Data Availability

Cryo-EM maps, masks, and structural coordinates have been deposited at the Research Collaboratory for Structural Bioinformatics (RCSB) and Electron Microscopy Data Bank (EMDB) and are publicly available as of the date of publication. Their accession codes are listed in the key resource table. Raw image data have been deposited at Mendeley and are publicly available as of data of publication. The DOI is listed in the key resource table. The mass spectrometry proteomics data have been deposited and will be publicly available at the ProteomeXchange Consortium via PRIDE^74^ partner repository and are also provided as Table S1 and S2. Their dataset identifier is listed in the key resource table. The paper does not report original code. Any additional information required to reanalyze the data reported in this paper is available from the lead contact upon request.
