# Supplementary material for: Non-canonical substrate recognition by the human WDR26-CTLH E3 ligase regulates prodrug metabolism
Source: Mol Cell. Author manuscript; Available in PMC 2024 Oct 16. (PMC7616709; doi:10.1016/j.molcel.2024.04.014)
Supplement: Supplementary figures [file EMS199317-supplement-Supplementary_figures.pdf]

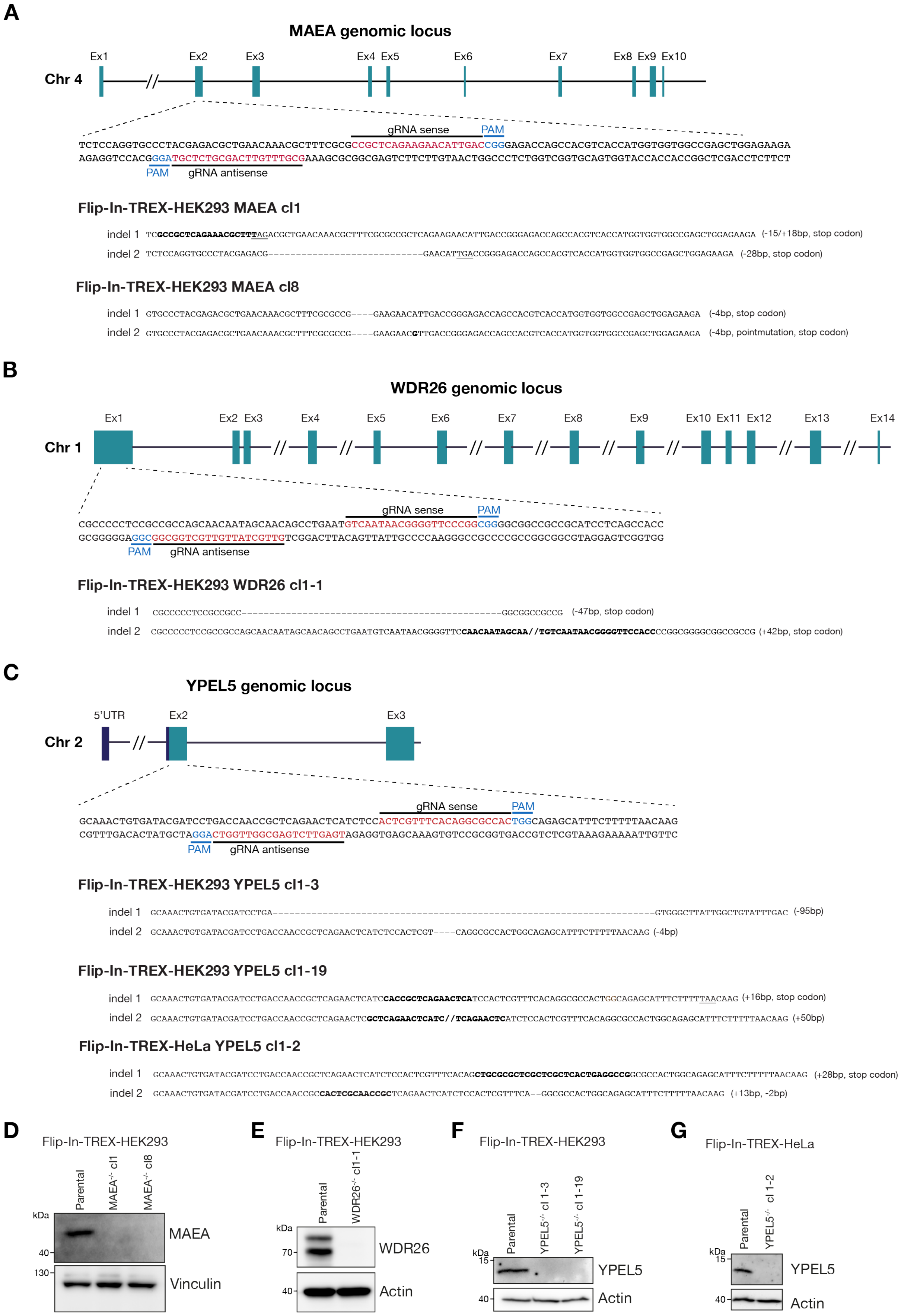
A
MAEA genomic locus
Chr 4
Ex1
Ex2
Ex3
Ex4
Ex5
Ex6
Ex7
Ex8
Ex9
Ex10
gRNA sense
PAM
PAM
gRNA antisense
Flip-In-TREX-HEK293 MAEA cl1
indel 1
(-15/+18bp, stop codon)
indel 2
(-28bp, stop codon)
Flip-In-TREX-HEK293 MAEA cl8
indel 1
(-4bp, stop codon)
indel 2
(-4bp, pointmutation, stop codon)
B
WDR26 genomic locus
Chr 1
Ex1
Ex2
Ex3
Ex4
Ex5
Ex6
Ex7
Ex8
Ex9
Ex10
Ex11
Ex12
Ex13
Ex14
gRNA sense
PAM
PAM
gRNA antisense
Flip-In-TREX-HEK293 WDR26 cl1-1
indel 1
(-47bp, stop codon)
indel 2
(+42bp, stop codon)
C
YPEL5 genomic locus
Chr 2
5'UTR
Ex2
Ex3
gRNA sense
PAM
PAM
gRNA antisense
Flip-In-TREX-HEK293 YPEL5 cl1-3
indel 1
(-95bp)
indel 2
(-4bp)
Flip-In-TREX-HEK293 YPEL5 cl1-19
indel 1
(+16bp, stop codon)
indel 2
(+50bp)
Flip-In-TREX-HeLa YPEL5 cl1-2
indel 1
(+28bp, stop codon)
indel 2
(+13bp, -2bp)
D
Flip-In-TREX-HEK293
Parental
MAEA-/- cl1
MAEA-/- cl8
kDa
40
130
MAEA
Vinculin
E
Flip-In-TREX-HEK293
Parental
WDR26-/- cl1-1
kDa
70
40
WDR26
Actin
F
Flip-In-TREX-HEK293
Parental
YPEL5-/- cl 1-3
YPEL5-/- cl 1-19
kDa
15
40
YPEL5
Actin
G
Flip-In-TREX-HeLa
Parental
YPEL5-/- cl 1-2
kDa
15
40
YPEL5
Actin

Figure S1. CRISPR-Cas9 (nickase) mediated knockout of human MAEA, WDR26, and YPEL5, related to Figure 1, 2 and 4

A) Schematic representation of genomic locus of *MAEA* showing exons structure, location of target guide RNAs (gRNAs) pairs, and PAM sequence. Insertions/deletions of each *MAEA* knockout clone of Flip-In TREX-HEK293 cells.

B) Schematic representation of genomic locus of *WDR26* showing exons structure, location of target guide RNAs (gRNAs) pairs, and PAM sequence. Insertions/deletions of *WDR26* knockout clone of Flip-In TREX-HEK293 cells.

C) Schematic representation of genomic locus of *YPEL5* showing exons structure, location of target guide RNAs (gRNAs) pairs, and PAM sequence. Insertions/deletions of each *YPEL5* knockout clone of Flip-In TREX-HEK293 and HeLa cells.

D-G) Immunoblot analysis of cell lysates of parental and knockout cell lines. Actin or Vinculin was used as loading control.

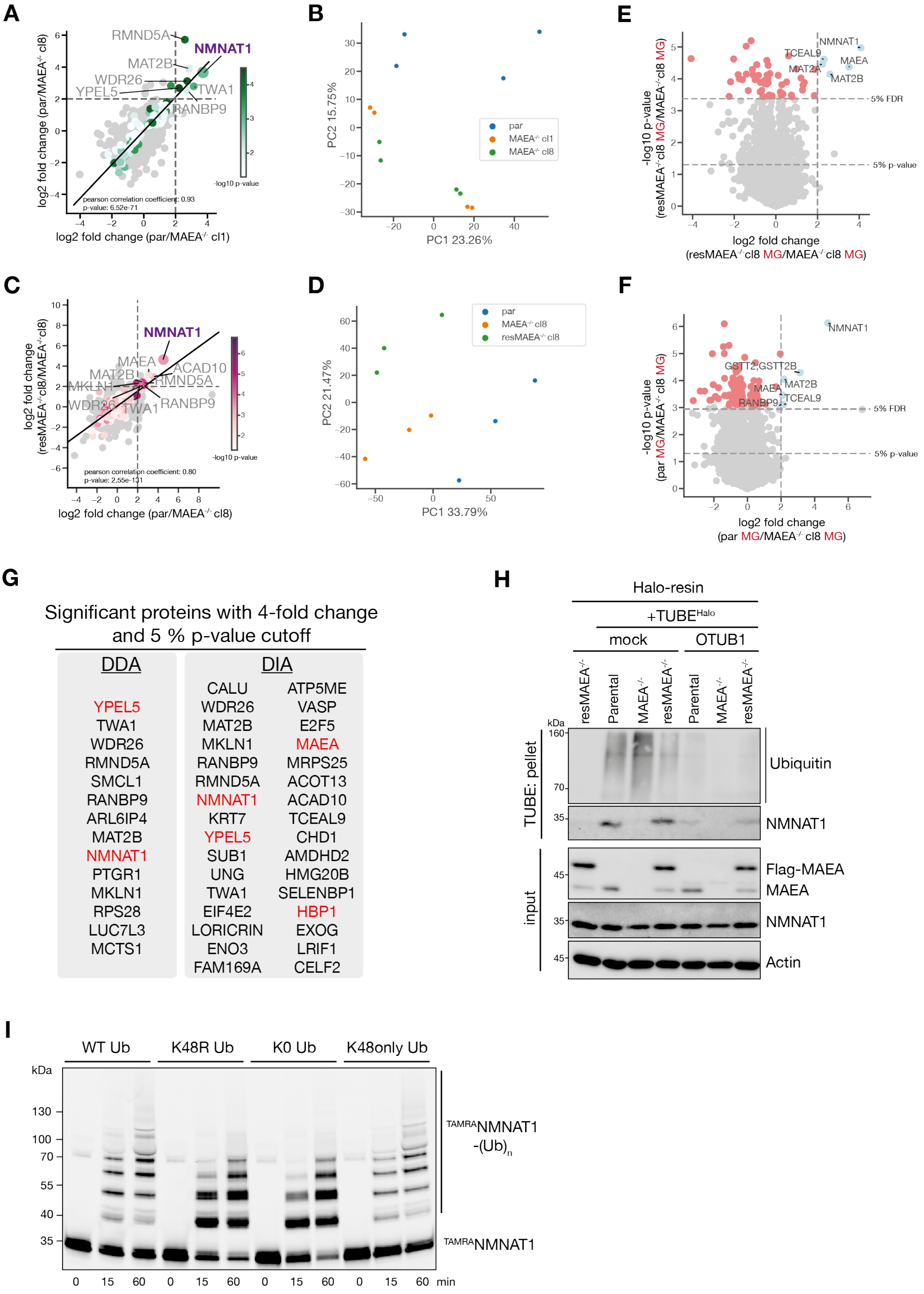

Figure S2. Ubiquitome-enrichment proteomics reveal a CTLH E3 substrate, related to Figure 1 and 2

A) Regression analysis of parental vs *MAEA*$^{-/-}$cl1 and parental vs *MAEA*$^{-/-}$cl8.

B) Principal component analysis of TUBE-enriched proteomes of Flip-In T-Rex-HEK293 parental (par), *MAEA*$^{-/-}$cl1, and *MAEA*$^{-/-}$cl8 cells with their biological replicas.

C) Regression analysis of *resMAEA*$^{-/-}$ vs *MAEA*$^{-/-}$cl8 and parental (par) vs *MAEA*$^{-/-}$cl8.

D) Principal component analysis of TUBE-enriched proteomes of Flip-In T-Rex-HEK293 parental, *MAEA*$^{-/-}$cl8, and *resMAEA*$^{-/-}$cl8 cells with their biological replicas.

E) Volcano plot of the -log10 p-values vs the log2 protein abundance of TUBE-precipitates in the presence of MG132 (MG) proteasomal inhibition between *resMAEA*$^{-/-}$ vs *MAEA*$^{-/-}$cl8. 5% p-value, 5% FDR and 4x cut off are indicated.

F) Volcano plot of the -log10 p-values vs the log2 protein abundance of TUBE-precipitates in the presence of MG132 (MG) proteasomal inhibition between parental vs *MAEA*$^{-/-}$cl8. 5% p-value, 5% FDR and 4x cut off are indicated.

G) Table summarizing significantly MAEA-regulated proteins (5% p-value and >4-fold change cut-off) from DDA and DIA approaches. Proteins with "G/KRKW" consensus basic motif are highlighted in red.

H) Lysates of indicated cell lines were either mock-treated or incubated with deubiquitylating enzyme OTUB1 prior to TUBE^{Halo} precipitation. Actin serves as protein input control.

I) Fluorescent scan of SDS-PAGE gel presenting time course of *in vitro* ubiquitylation of N-terminally fluorescently tagged TAMRA-NMNAT1 (^{TAMRA}NMNAT1) by WDR26-CTLH E3 comparing indicated ubiquitin (Ub) variants (wildtype (WT), K48R mutated (K48R), all K to R mutated (K0), and all K mutated to R except K48 (K48only)).

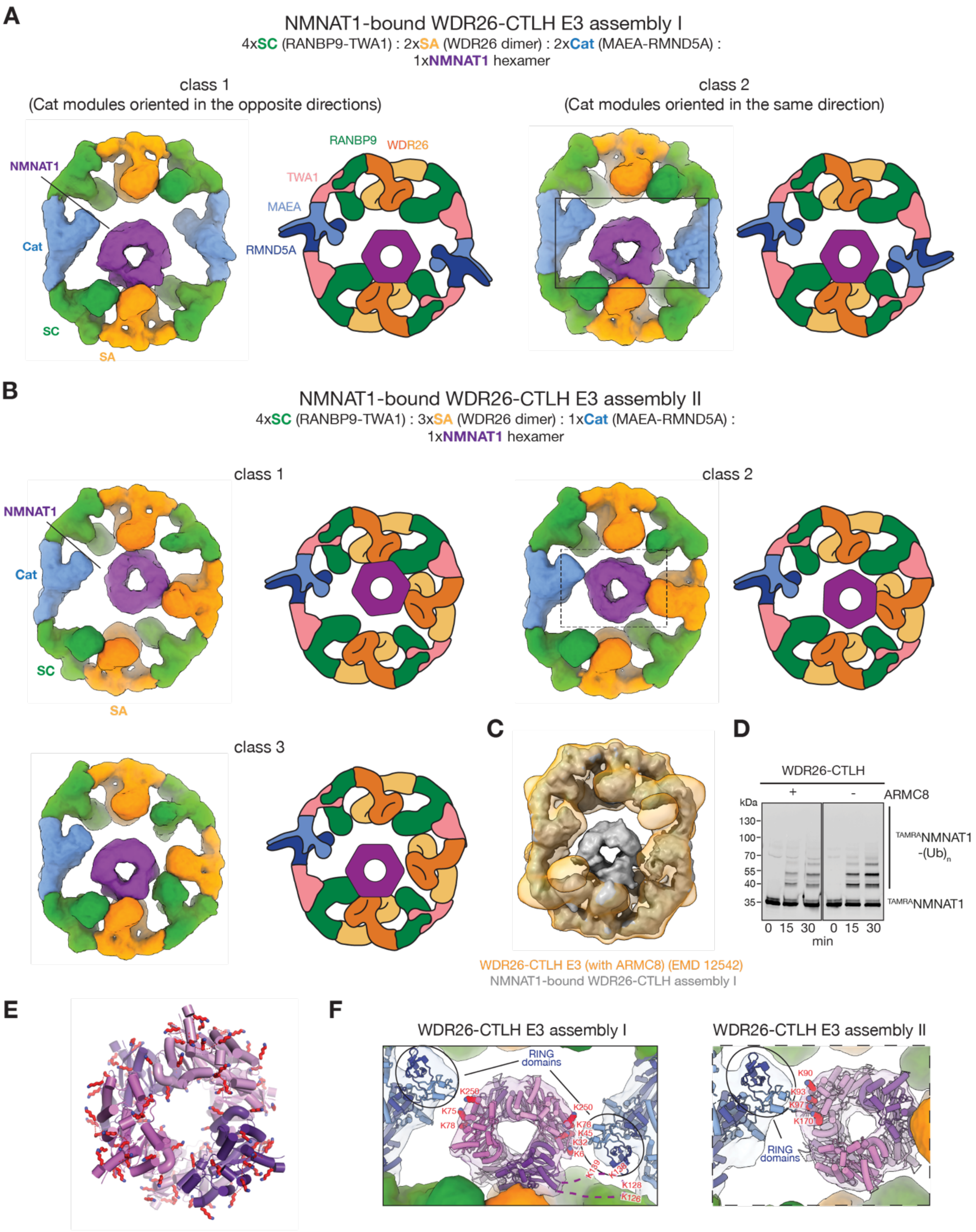

Figure S3. Structures of WDR26-CTLH E3 assemblies capturing NMNAT1, related to Figure 3

A, B) Comparison of the architecture and the mode of NMNAT1 capture by WDR26-CTLH E3 assembly I and II. For each class, segmented cryo-EM maps highlight differing module compositions (left, color-coded by a given CTLH E3 module or NMNAT1), whereas the

cartoons (right) depict the constituent subunits. The module and subunit stoichiometries for assemblies I and II are listed above the corresponding maps and cartoons.

C) Overlay of the prior map of the ARMC8-containing WDR26-CTLH E3 (EMD-12542, orange) and the NMNAT1-bound WDR26-CTLH E3 assembly I (grey) illustrating their overall similarity.

D) *In vitro* assay comparing ubiquitylation of N-terminally TAMRA-labelled NMNAT1 (^{TAMRA}NMNAT1) by WDR26-CTLH E3 complex expressed and purified with or without ARMC8. NMNAT1 was visualized by a fluorescent scan of SDS-PAGE gel.

E) Crystal structure of NMNAT1 (PDB: 1KQN, protomers colored in different shades of violet and pink) highlighting its numerous solvent-exposed lysine residues (red sticks).

F) Close-ups of representative maps of assembly I and II (marked in A and B) fit with NMNAT1 crystal structure (PDB: 1KQN) and AlphaFold2 model of RMND5A-MAEA illustrating the differing orientations of NMNAT1 core with respect to the RING domains (black ovals). Such arrangements would potentially enable targeting multiple NMNAT1 lysines (red sticks). The NMNAT1 loop is represented as a dashed line (left).

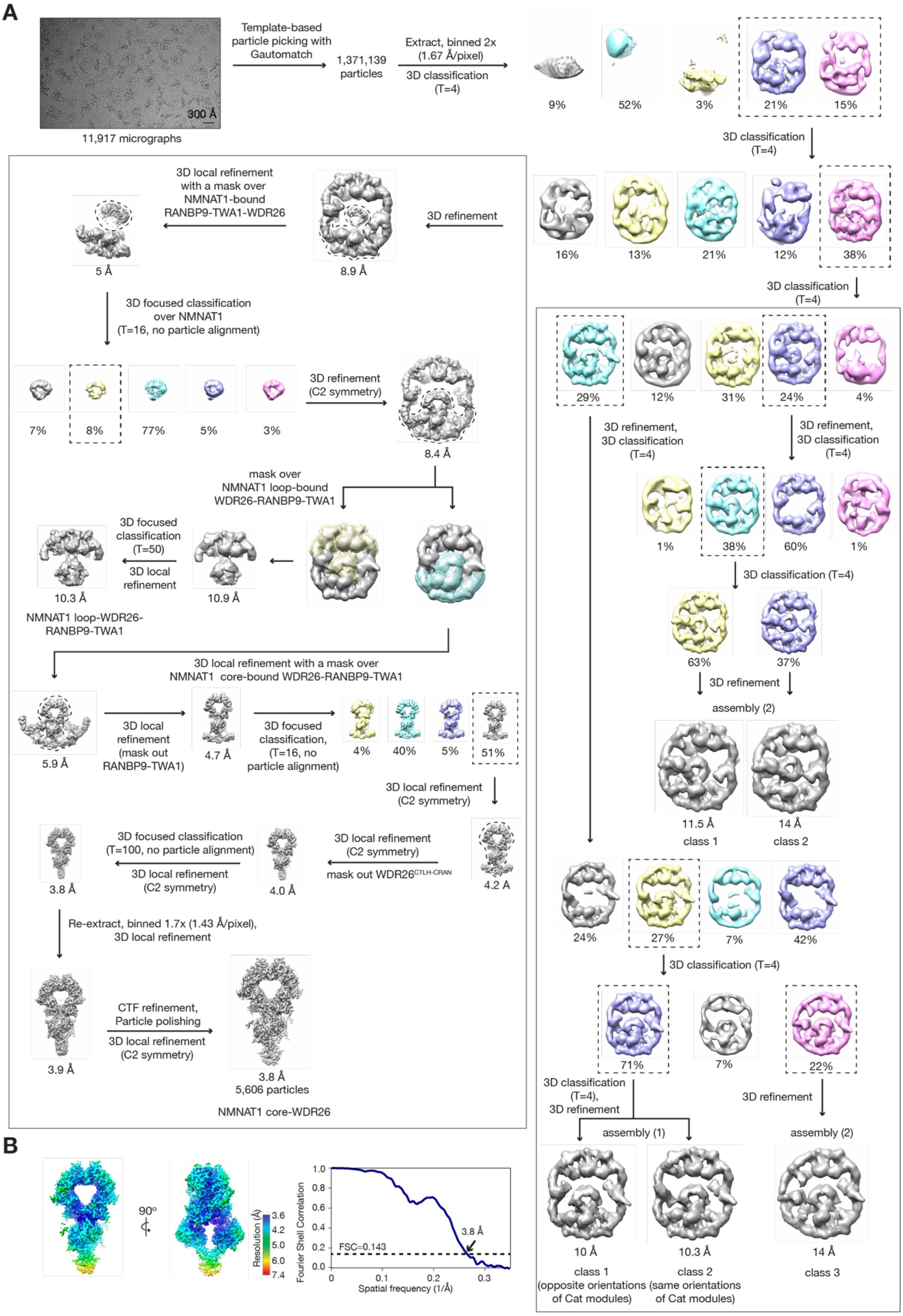

A
300 Å
11,917 micrographs
Template-based particle picking with Gautomatch
1,371,139 particles
Extract, binned 2x (1.67 Å/pixel)
3D classification (T=4)
9%
52%
3%
21%
15%
3D classification (T=4)
16%
13%
21%
12%
38%
3D classification (T=4)
29%
12%
31%
24%
4%
3D refinement, 3D classification (T=4)
3D refinement, 3D classification (T=4)
1%
38%
60%
1%
3D classification (T=4)
63%
37%
3D refinement
assembly (2)
11.5 Å
class 1
14 Å
class 2
24%
27%
7%
42%
3D classification (T=4)
71%
7%
22%
3D classification (T=4), 3D refinement
3D refinement
assembly (1)
assembly (2)
10 Å
class 1 (opposite orientations of Cat modules)
10.3 Å
class 2 (same orientations of Cat modules)
14 Å
class 3
3D refinement
3D local refinement with a mask over NMNAT1-bound RANBP9-TWA1-WDR26
5 Å
8.9 Å
3D focused classification over NMNAT1 (T=16, no particle alignment)
7%
8%
77%
5%
3%
3D refinement (C2 symmetry)
8.4 Å
mask over NMNAT1 loop-bound WDR26-RANBP9-TWA1
3D focused classification (T=50)
3D local refinement
10.3 Å
10.9 Å
NMNAT1 loop-WDR26-RANBP9-TWA1
3D local refinement with a mask over NMNAT1 core-bound WDR26-RANBP9-TWA1
5.9 Å
3D local refinement (mask out RANBP9-TWA1)
4.7 Å
3D focused classification, (T=16, no particle alignment)
4%
40%
5%
51%
3D local refinement (C2 symmetry)
4.2 Å
3D local refinement (C2 symmetry)
mask out WDR26CTLH-CRAN
4.0 Å
3D focused classification (T=100, no particle alignment)
3D local refinement (C2 symmetry)
3.8 Å
Re-extract, binned 1.7x (1.43 Å/pixel), 3D local refinement
3.9 Å
CTF refinement, Particle polishing
3D local refinement (C2 symmetry)
3.8 Å
5,606 particles
NMNAT1 core-WDR26
B
90°
Resolution (Å)
3.6
4.2
5.0
6.0
7.4
Fourier Shell Correlation
1.0
0.8
0.6
0.4
0.2
0.0
FSC=0.143
3.8 Å
0
0.1
0.2
0.3
Spatial frequency (1/Å)

Figure S4. Details of cryo-EM data processing of NMNAT1-bound WDR26-CTLH E3 dataset, related to Figure 3

A) Simplified flow chart of high-resolution data processing yielding low-resolution maps of multiple classes of NMNAT1-bound WDR26-CTLH E3 assembly I and II as well as a high-resolution focused-refined map of WDR26 dimer capturing NMNAT1 core, which permitted building a structural model. The scale bar in the representative micrograph corresponds to 300 Å.

B) Gold-standard Fourier shell correlation (FSC) plot (right). The dotted line represents 0.143 cut-off criterion for estimating nominal resolution. The final map is color-coded to illustrate variations in its local resolution (left).

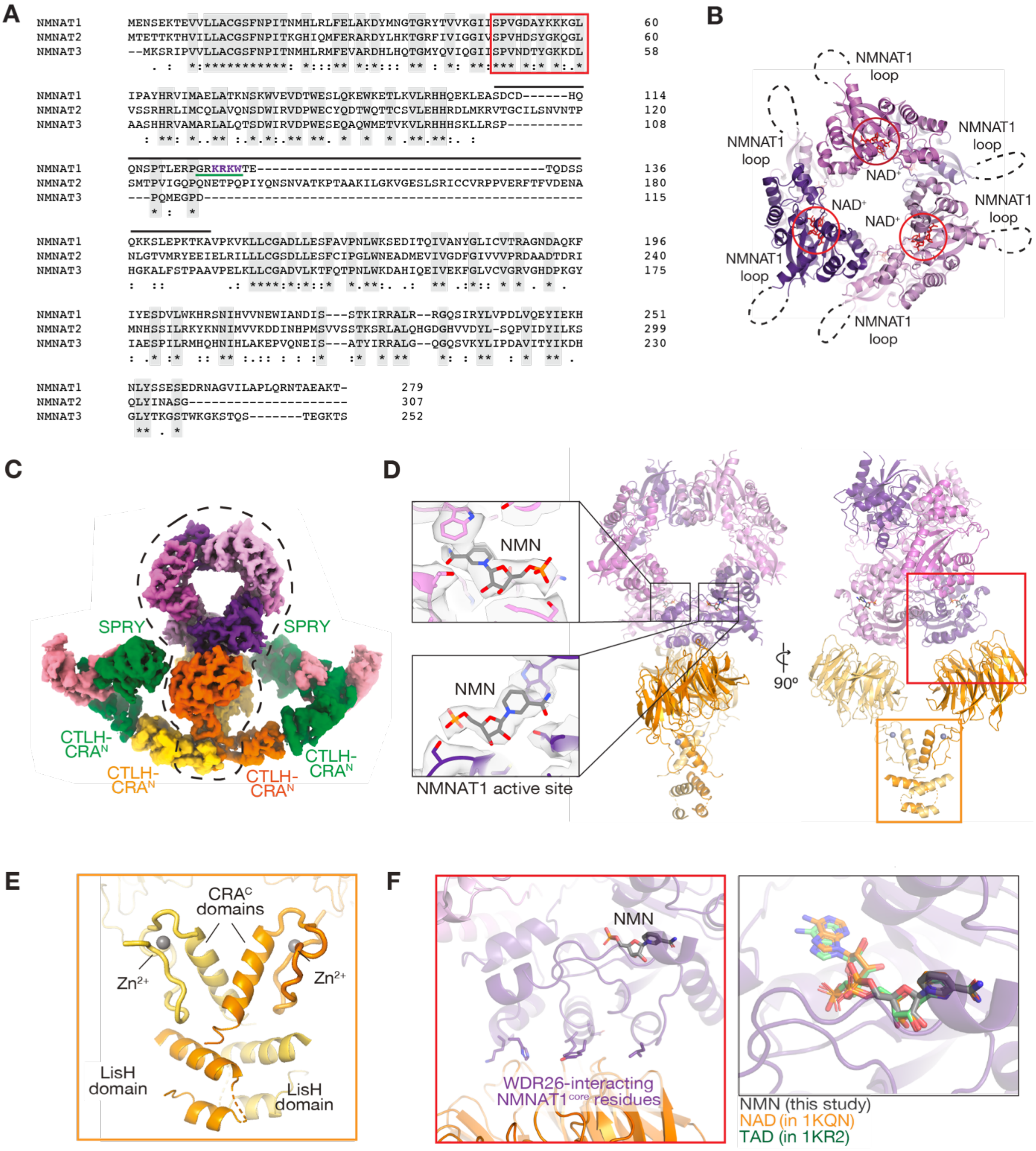

Figure S5. Structural features of NMNAT1 and its complex with WDR26-CTLH E3, related to Figure 3

A) Multiple sequence alignment of NMNAT1, NMNAT2 and NMNAT3 highlighting their NAD$^+$ binding site (red box), and disordered loops (black line). The loop from NMNAT1 harbors the WDR26-binding basic motif (NMNAT1^{basic}, violet) and the nuclear localization sequence (NLS, green).

B) Crystal structure of NMNAT1 hexamer (PDB: 1KQN) in complex with NAD^+ highlighting its active sites (red circle) and the flexible loops (dotted lines) protruding from folded domains of each protomer.

C) Segmented focused refined map (color-coded as in Figure 3A) highlighting the mode of WDR26 dimer incorporation into the complex through interactions of its CTLH-CRA^N with the corresponding domains from RANBP9 and contacts between RANBP9 SPRY and WDR26 β-propellers. The dashed line represents the mask used for focused refinement yielding the high-resolution map of NMNAT1 core-bound WDR26 (shown in Fig. 3C).

D) Structure of NMNAT1 core-bound WDR26 highlighting NMN-bound NMNAT1 active site. The two NMNAT1 cores that lodge on WDR26 propellers contain a clear NMN density (extracted from the focused-refined map from Figure 3C and colored grey in the close-up; the fitted NMN coordinates are displayed as sticks). The orange and red boxes indicate the WDR26 homodimerization domain (shown in E), and the interface between the WDR26 β-propeller and the globular domain of NMNAT1 (shown in F).

E) Close-up showcasing CRA^C and LisH domains of WDR26 responsible for its homodimerization. Zn^{2+} ions, which structure the regions downstream of the CRA^C helices, are indicated as gray spheres.

F) Close-up showing the position of NMN bound to the NMNAT1 active site, which is distal to the WDR26-binding interface (the WDR26 β-propeller-interacting NMNAT1 residues are shown as sticks). The position of NMN corresponds to that of NAD^+ and NAD^+-mimetic prodrug TAD extracted from the superposed crystal structures of their complexes with NMNAT1 (PDB 1KQN and 1KR2).

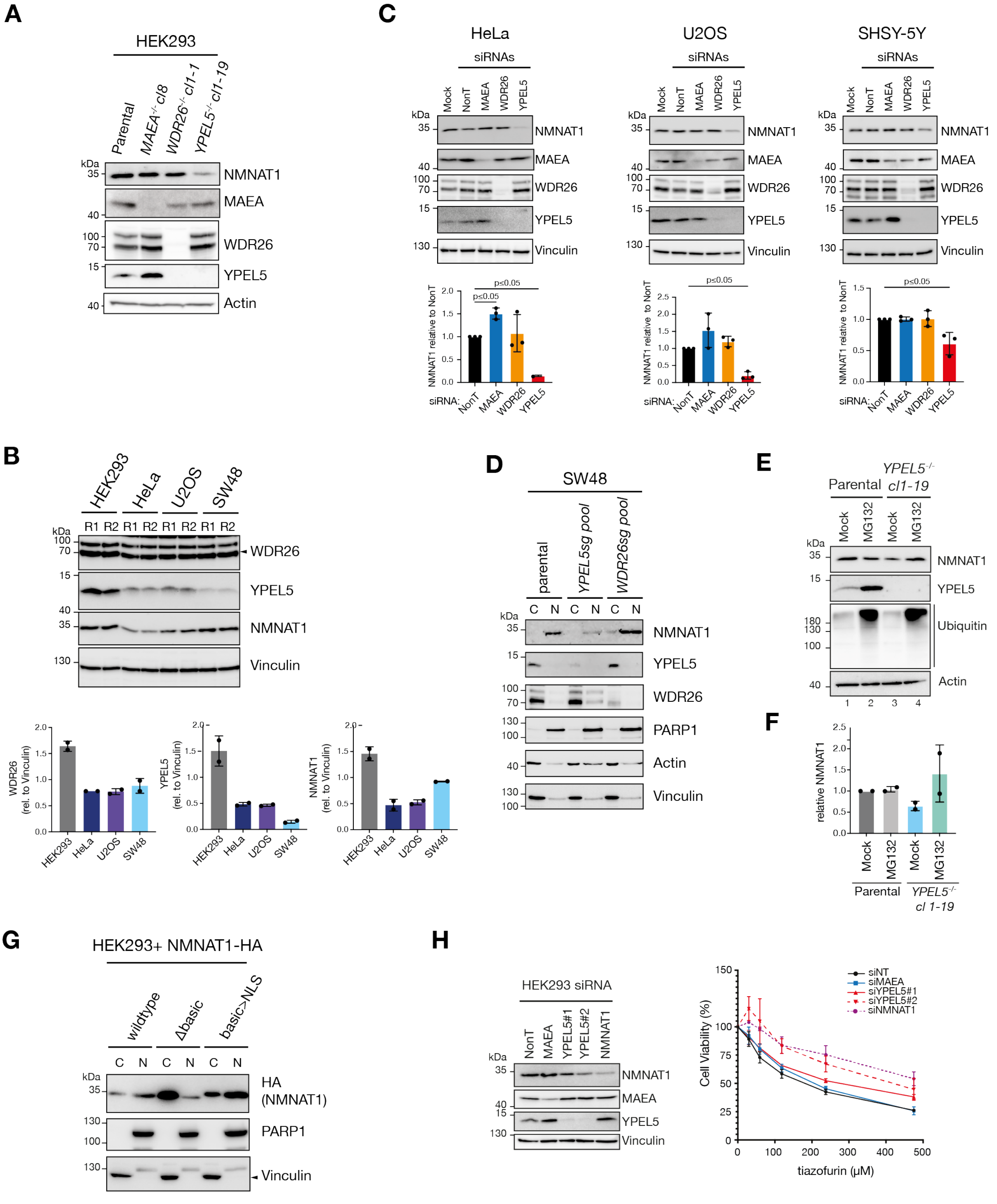

Figure S6 YPEL5-WDR26-CTLH E3 modulates NMNAT1 amounts, related to Figure 4 and 5

A) Immunoblot analysis of whole cell lysates from HEK293 parental and CRISPR-Cas9 knockout cell lines. Actin serves as input control.

B) Immunoblot analysis of whole cell lysates of indicated cell lines (two biological repetitions R1 and R2, top panels). Quantitation of immunoblot signals of WDR26, YPEL5, and NMNAT1 normalized to Vinculin (bottom panels). Graph shows results by means +/- SD of n=2.

C) Indicated cell lines were mock treated or subjected to siRNA targeting *MAEA*, *WDR26*, or *YPEL5,* and whole cell lysates analyzed by immunoblot (top panels). NonT, non-targeting siRNA control. Quantitation of NMNAT1 immunoblot signals normalized to Vinculin and relative to NonT control values (bottom panels). Graph shows results by means +/- SD of n=3.

D) SW48 cells were CRISPR-Cas9-edited to targeting of *YPEL5* (*YPEL5sg pool*) and *WDR26* (*WDR26sg pool*), and cell lysates fractionated into cytosolic (C) and nuclear (N) fractions followed by immunoblot analysis. PARP1 serve as nuclear, Actin and Vinculin as cytosolic makers.

E) HEK293 parental and *YPEL5*$^{-/-}$ cells were either mock-treated or treated with the proteasomal inhibitor MG132 (10 μM) overnight and cell lysates analysed by immunoblot.

F) Quantitation of NMNAT1 immunoblot signals from E) normalized to Actin and relative to mock-treated parental values. Graph shows results by means +/- SD of n=2.

G) HEK293 cells were transiently transfected with NMNAT1-HA wildtype, NMNAT1-HA with basic motif deletion (Δbasic), and NMNAT1-HA with basic motif to NLS replacement (basic>NLS), cell lysates fractionated into cytosolic (C) and nuclear (N) fractions, and analysed by immunoblot analysis. Actin and PARP1 serve as cytosolic and nuclear makers, respectively.

H) HEK293 cells were subjected to siRNA targeting *MAEA*, *YPEL5*, and *NMNAT1*, treated with tiazofurin (0-475 μM), and cell viability assessed after 96 hours. Efficiency of siRNA was assessed by immunoblot analysis of cell lysates (left). Graph of cell viability (%) (right) shows results by means +/- SD of n=3.

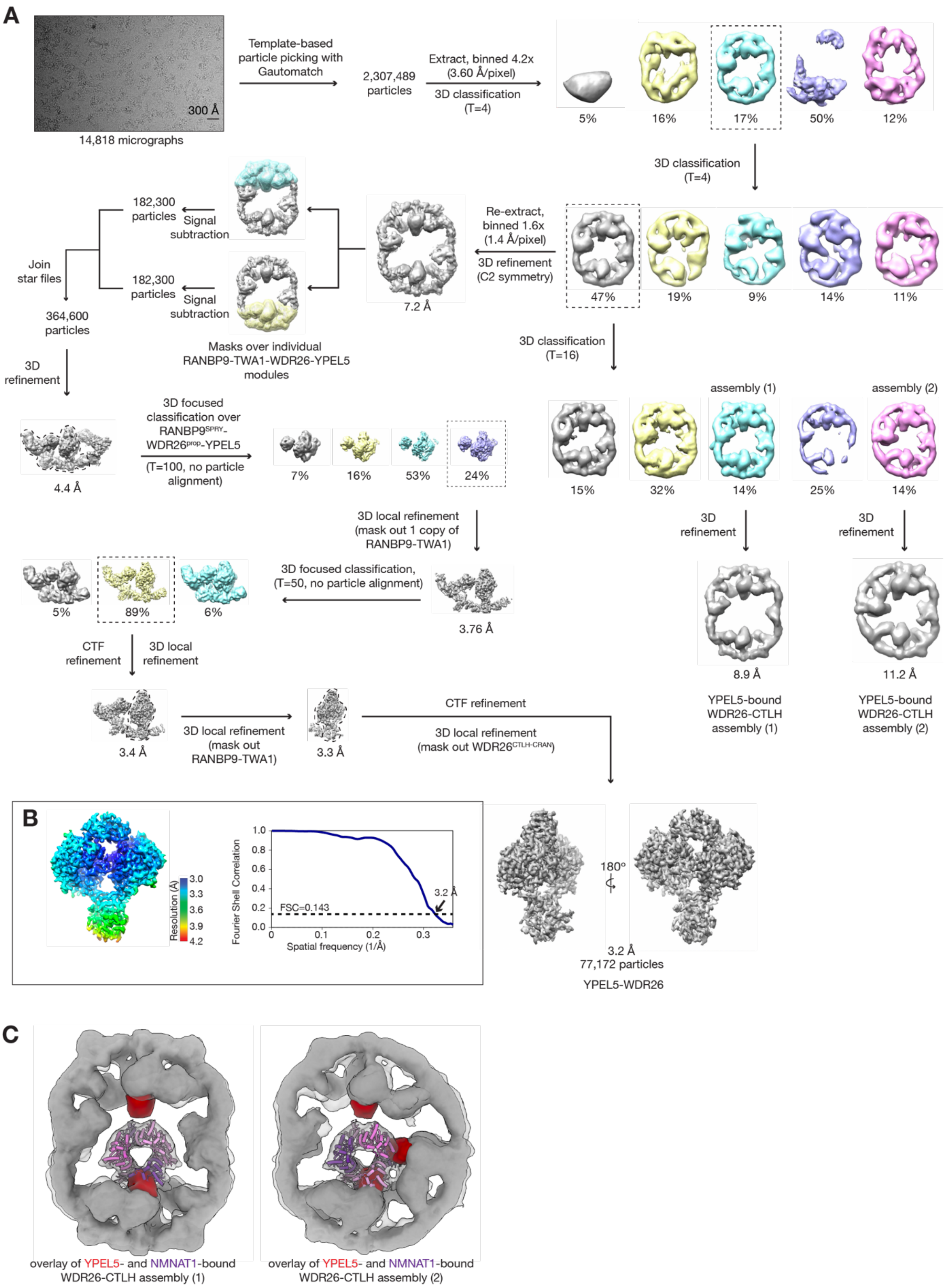

Figure S7. Details of cryo-EM data processing of YPEL5-bound WDR26-CTLH E3 dataset, related to Figure 6

A) Simplified flow chart of high-resolution data processing yielding low-resolution maps of YPEL5-bound WDR26-CTLH E3 assembly I and II as well as a high-resolution focused-refined map of WDR26 dimer engaging YPEL5, which permitted building a structural model. The scale bar in the representative micrograph corresponds to 300 Å.

B) Gold-standard Fourier shell correlation (FSC) plot (right). The dotted line represents 0.143 cut-off criterion for estimating nominal resolution. The final map is color-coded to illustrate variations in its local resolution (left).

C) Overlay of low-resolution maps showing steric clashes between NMNAT1 and YPEL5 bound to both WDR26-CTLH E3 assembly I (left) and II (right).

Table S3. Cryo-EM data collection, refinement and validation statistics, related to Figure 3 and 6

	NMNAT1-bound WDR26-CTLH E3 assembly I		NMNAT1-bound WDR26-CTLH E3 assembly II			RANBP9-TWA1-WDR26 module binding NMNAT1 core (EMD-18172)	RANBP9-TWA1-WDR26 module binding NMNAT1 loops (EMD-18173)	Structure of NMNAT1-bound WDR26 dimer (EMD-18345) (PDB 8QE8)	YPEL5-bound WDR26-CTLH E3		Structure of YPEL5-bound WDR26 dimer (EMD-18316) (PDB 8QBN)
	class 1 (EMD-18174)	class 2 (EMD-18175)	class 1 (EMD-18176)	class 2 (EMD-18177)	class 3 (EMD-18178)				assembly I (EMD-18170)	assembly II (EMD-18171)	
**Data collection and processing**											
Microscope/detector	Titan Krios/K3								Titan Krios/K3		
Magnification	105,000								105,000		
Voltage (kV)	300								300		
Electron exposure ($e^{-}/Å^2$)	71.4								71.2		
Defocus range (μm)	0.8 – 2.3								0.8 – 2.3		
Pixel size (Å)	0.851								0.851		
Symmetry imposed	C_1					C_2			C_1		
Initial particle images (no.)	1,371,139								2,307,489		
Final particle images (no.)	4,590	6,146	6,420	3,747	3,454	5,826	6,694	5,606	26,720	24,712	77,172
Map resolution (Å) (FSC threshold: 0.143)	10.0	10.3	11.5	13.9	12.2	4.7	8.7	3.8	8.9	11.2	3.2
Map resolution range (Å)								3.6-7.4			3.0-4.2
**Refinement**											
Initial model used (PDB code)								1GZU AlphaFold2			AlphaFold2
Model resolution (Å) (FSC threshold: 0.143)								3.8			3.2
Map sharpening								-50			-104

B factor (Å^2)			
Model composition			
Non-hydrogen atoms	15,796		7,021
Protein residues	2,044		897
Ligands	2 Zn^{2+}, 2 NMN		3 Zn^{2+}
B factors (Å^2)			
Protein	23.2		39.4
Ligand	25.3		63.2
R.m.s. deviations			
Bond lengths (Å)	0.004		0.004
Bond angles (°)	0.602		0.589
Validation			
MolProbity score	1.6		1.5
Clashscore	5.5		4.5
Poor rotamers (%)	0.1		0.3
Ramachandran plot			
Favored (%)	95.9		95.7
Allowed (%)	4.1		4.3
Disallowed (%)	0		0
