# Supplementary material for: Non-canonical substrate recognition by the human WDR26-CTLH E3 ligase regulates prodrug metabolism
Source: Mol Cell. Author manuscript; Available in PMC 2024 Oct 16. (PMC7616709; doi:10.1016/j.molcel.2024.04.014)
Supplement: Supplementary Tables [file EMS199317-supplement-Supplementary_Tables.docx]

**Table S1: List of *MAEA*-dependent proteome in HEK293 cells identified by Data-dependent acquisition (DDA) MS analysis, related to Figure 1B**

**Table S2: List of *MAEA*-dependent proteome in HEK293 cells identified by Data-independent acquisition (DIA) MS analysis, related to Figure 1C**
